# Proteomic Profiling of *Burkholderia cenocepacia* Clonal Isolates with Different Virulence Potential Retrieved from a Cystic Fibrosis Patient during Chronic Lung Infection

**DOI:** 10.1371/journal.pone.0083065

**Published:** 2013-12-13

**Authors:** Andreia Madeira, Sandra C. dos Santos, Pedro M. Santos, Carla P. Coutinho, Jean Tyrrell, Siobhán McClean, Máire Callaghan, Isabel Sá-Correia

**Affiliations:** 1 Institute for Biotechnology and Bioengineering, Centre for Biological and Chemical Engineering, Department of Bioengineering, Instituto Superior Técnico, Universidade de Lisboa, Lisbon, Portugal; 2 Centre of Microbial Host Interactions, Department of Science, ITT-Dublin, Dublin, Ireland; Quuen's University Belfast, United Kingdom

## Abstract

Respiratory infections with *Burkholderia cepacia* complex (Bcc) bacteria in cystic fibrosis (CF) are associated with a worse prognosis and increased risk of death. In this work, we assessed the virulence potential of three *B. cenocepacia* clonal isolates obtained from a CF patient between the onset of infection (isolate IST439) and before death with cepacia syndrome 3.5 years later (isolate IST4113 followed by IST4134), based on their ability to invade epithelial cells and compromise epithelial monolayer integrity. The two clonal isolates retrieved during late-stage disease were significantly more virulent than IST439. Proteomic profiling by 2-D DIGE of the last isolate recovered before the patient’s death, IST4134, and clonal isolate IST439, was performed and compared with a prior analysis of IST4113 vs. IST439. The cytoplasmic and membrane-associated enriched fractions were examined and 52 proteins were found to be similarly altered in the two last isolates compared with IST439. These proteins are involved in metabolic functions, nucleotide synthesis, translation and protein folding, cell envelope biogenesis and iron homeostasis. Results are suggestive of the important role played by metabolic reprogramming in the virulence potential and persistence of *B. cenocepacia*, in particular regarding bacterial adaptation to microaerophilic conditions. Also, the content of the virulence determinant AidA was higher in the last 2 isolates. Significant levels of siderophores were found to be secreted by the three clonal isolates in an iron-depleted environment, but the two late isolates were more tolerant to low iron concentrations than IST439, consistent with the relative abundance of proteins involved in iron uptake.

## Introduction

Long-term respiratory infections with *Burkholderia cepacia* complex (Bcc) bacteria in cystic fibrosis (CF) patients generally lead to an accelerated decline in lung function and, in many cases, to a fatal necrotizing pneumonia known as cepacia syndrome [1,2]. These bacteria are serious opportunistic pathogens and virtually impossible to eradicate from the CF lung [1,3,4]. Moreover, Bcc species are characterised by remarkable genome plasticity, providing them with a major advantage to adapt to the highly stressful CF lung environment [5–7]. Widespread positive selection pressure across the genome of Bcc bacteria leads to the emergence of multiple phenotypic clonal variants that exhibit different antimicrobial susceptibility patterns, as well as other phenotypic alterations relevant in the context of bacterial pathogenesis [8–11]. 

 Understanding the underlying mechanisms employed by Bcc bacteria to adapt to the CF airway environment under such conditions is crucial to deal with chronic infections and improve disease management. We have recently contributed to the elucidation of these adaptive mechanisms based on extensive phenotypic, genotypic and genome-wide expression analysis of selected *B. cenocepacia* clonal variants [7,8,12,13]. These are part of an extensive collection comprising over 700 clinical isolates of different Bcc species, gathered during an 18-year long epidemiological survey of Bcc bacteria involved in respiratory infections at the major Portuguese CF Treatment Centre at Santa Maria Hospital in Lisbon [7,14,15]. Recent studies have focused on a series of 11 *B. cenocepacia* clonal isolates (*recA* lineage III-A) retrieved from the sputum of a chronically colonised CF patient (J), from the onset of infection to his death 3.5 years later with cepacia syndrome, where clonal expansion of the first isolate was proposed to occur [8,14]. Three isolates from the set of 11 variants have been particularly scrutinised by genome-wide expression analyses [12,13]: IST439, the first *B. cenocepacia* isolate recovered, which presumably initiated the infection; IST4113, obtained almost 3 years later after a period of exacerbated infection and intravenous therapy; and IST4134, the last isolate retrieved from the patient immediately before death with cepacia syndrome. 

 The study of mechanisms and dynamics of microbial evolution within a host environment, and how it relates to pathogenicity and virulence, is currently a topic of paramount importance in health sciences. In this study, we examined the epithelial interactions of clonal isolates IST439, IST4113, and IST4134, and found that the 2 isolates retrieved during late-stage disease have a significantly greater capacity for epithelial invasion than the one recovered at the onset of infection. In an effort to identify mechanisms underlying this altered virulence phenotype, we employed a 2-D Difference Gel Electrophoresis (2-D DIGE) quantitative proteomics approach, under the assumption that proteins that are similarly altered in both IST4113 and IST4134 compared to IST439 are likely to be involved in the increased persistence of those clonal isolates in the CF lung. To this end, we compared the proteomes of IST439 and IST4134 (this work) and integrated these findings with the prior analysis of IST4113 *vs.* IST439 [13], which had been mainly focused on the identification of mechanisms of genetic adaptation leading to antimicrobial resistance in the CF airways. The results highlight the involvement of proteins associated with metabolic functions in increased persistence and virulence potential, in agreement with recent genome-wide studies [16–18].

## Materials and Methods

### *Burkholderia cenocepacia* isolates and growth conditions

The three clinical *B. cenocepacia* isolates studied, IST439, IST4113 and IST4134, were recovered from the sputum of a CF patient under surveillance at the CF Centre of Hospital de Santa Maria [7,14,15]. The clonal isolates were obtained between January 1999 and July 2002. Studies involving the use of these isolates were approved by the ethics committee of the Hospital (Comissão de Ética para a Saúde do HSM/FML), and the anonymity of the patients is preserved. No unpublished clinical data is included in this study. Studies regarding retrospective molecular microbiology analyses and functional genomics were authorized by the ethics committee. The specific samples used in this study have also been described in previous publications [7,14,15]. For the proteomic analysis, cell samples of *B. cenocepacia* isolates were prepared by suspending well isolated colonies from Luria Bertani (LB, Difco, Sparks, MD USA) agar plates in 3 mL LB broth, followed by overnight growth at 37°C with shaking at 250 rpm. These cultures were used to obtain cells in mid-exponential phase (OD_640 nm_ of 0.4±0.05). After dilution to a standardised OD_640 nm_ of 0.2 in NaCl 0.9% (w/v), 100 µL of these cell suspensions were plated onto LB agar plates and incubated for 24 hours at 37°C.

### Transepithelial resistance of bronchial epithelial monolayers exposed to *B. cenocepacia* clonal variants

Polarized epithelial monolayers were established using two independent bronchial epithelial cells, which expressed functional CFTR (16HBE14o-) or were homozygous negative for the ΔF508 mutation (CFBE41o-) (a generous gift from Dr. Dieter Gruenert, University of California, San Francisco). 16HBE14o- cells [19] were seeded onto 0.3 µM permeable filter supports at a density of 1×10^5^ cells per mL with a liquid/liquid interface for 6 days. CFBE41o- cells (CFTR negative) [20,21] were seeded onto 0.3 µM filters at a density of 7×10^5^ cells per mL with a liquid/liquid interface for one day and changed to air/liquid interface for five days. On day five, antibiotic was removed from media and transepithelial resistance (TER) measurements taken using electrodes (World Precision Instruments), to confirm monolayer integrity. Bacterial strains were grown in MM9 media and on day 6, the bacteria were added to the epithelial monolayers at a Multiplicity of infection (MOI) of 50:1. The TERs of both control (either no bacteria added to the culture or *Escherichia coli*-exposed monolayers) and *B. cenocepacia*-exposed monolayers were measured at 0, 2, 4, 6, 8, 12 and 24 hours. TER was calculated as follows: (Resistance of cells on filter – resistance of blank filters) × 1.13 cm^2^.

 Data represents the average of three separate experiments and error bars represent the standard error of the mean. Statistical analysis was carried out using a one-way ANOVA and Holms-Sidak post-test. 

### Invasion of *B. cenocepacia* sequential clonal variants into human lung epithelial cells

CFBE41o- (CFTR negative) and 16HBE14o- (CFTR expressing) cells were seeded into 24 well plates at a density of 4×10^5^ cells per well in antibiotic free media for 24 hours at 37°C in a 5% CO_2_ humidified atmosphere. Medium was removed and cells were washed three times with Phosphate Buffered Saline (PBS). Strains were grown overnight in LB media and 4×10^5^ CFU/mL were added to Minimum Essential Medium (MEM) alone. The bacterial cultures were added to the wells at an MOI of 10:1 and the plate was centrifuged at 700*g* for five minutes to allow bacterial interaction/ attachment to epithelial cells. Cells and bacteria were incubated for two hours to allow invasion. The supernatant was replaced with 1 mL of a highly inhibitory (2 mg/mL) ciprofloxacin solution and incubated for a further two hours to kill the extracellular bacteria. Cells were washed three times vigorously with PBS and 500 µL of cell lysis buffer (PBS, 10 mM EDTA, 0.25% (w/v) Triton X-100) was added for 20 min. at room temperature. The resulting lysate was serially diluted in Ringer’s solution and quantified by viable counts on LB agar after 48 hours.

 The effectiveness of the antibiotic treatment in killing the extracellular bacteria was confirmed by plating the cellular washes following antibiotic treatment onto LB agar plates and incubating for 48 hours. No growth was detected on these plates. Data represents the average of three separate experiments and error bars represent the standard error of the mean. Statistical analysis was carried out using a one-way ANOVA and Holms-Sidak post-test.

### Quantitative proteomic analysis based on 2-D DIGE

A 2-Dimensional Difference Gel Electrophoresis (2-D DIGE) quantitative proteomic approach was used to compare the cytoplasmic and the membrane-associated protein enriched fractions of *B. cenocepacia* clinical isolates IST439 and IST4134. The analysis was performed as previously described [13]. As before, bacterial cells were harvested from solid LB agar plates instead of planktonic cultures, because growth on the solid agar surface is believed to be a more accurate reflection of the physiological conditions experienced by *B. cenocepacia* in the airways of CF patients. Briefly, cells were washed from 8 LB-agar plates for each *B. cenocepacia* isolate, independently prepared, with 0.9% NaCl and resuspended in lysis buffer (10 mM Tris base, 100 mM sucrose Halt^TM^ protease inhibitors cocktail (Pierce, Rockford, USA)). Cell disruption was carried out by sonication and the supernatant (crude extract) was fractionated into cytoplasmic and membrane-associated protein fractions by ultracentrifugation at 100,000*g*, during 90 min., at 4°C. The supernatant obtained was enriched in the cytoplasmic protein fraction while the pellet corresponded to the membrane-associated protein enriched fraction. The membrane-associated protein fraction was washed twice with lysis buffer followed by a second ultracentrifugation (100,000*g*, 60 min., 4°C) to reduce cytoplasmic protein contaminants. The membrane-associated protein fraction was resuspended in lysis buffer containing 2% dodecylmaltoside. Protein concentration in the extracts was quantified using a Bicinchoninic Acid quantification kit (Pierce), and 50 µg aliquots of the protein samples were subjected to a clean-up process with the 2-D Clean-up kit (GE Healthcare, Uppsala, Sweden). Fluorescent labelling of proteins with Cy-dye™ (GE Healthcare), isoelectric focusing (IEF), protein separation by molecular weight, gel scanning and analysis, and preparation of gels for posterior protein identification, were also performed as previously described [13]. The software package Progenesis Samespots (Nonlinear Dynamics) was used to automatically detect protein spots and normalise individual spot volumes against total spot volumes for a given gel. Average normalised levels for each isolate were then compared using one-way ANOVA between-group test. Only statistically significant spots (P < 0.05) were selected for analysis. Differential expression between isolates IST439 and IST4134 was determined and a threshold of at least a 1.5-fold increase or 0.67-fold decrease between averaged gels was considered. Spots that showed evidence of saturation were not further analysed. The results represent two independent growth experiments and three technical replicates, in a total of six replicates per sample.

 Identification of protein spots of interest was performed by mass spectrometry (MS) in the proteomic unit at Centro Nacional de Investigaciones Cardiovasculares Carlos III, Madrid, Spain (CNIC Foundation), as detailed before [13]. The classification into functional categories was performed using the *Burkholderia* Genome Database (http://www.burkholderia.com) [22], TIGR database (http://www.tigr.org/), the Role Category Lists, KEGG database (http://www.genome.jp/kegg/) and NCBI (http://www.ncbi.nlm.nih.gov/). The mRNA levels of a randomly selected set of genes, encoding proteins with altered expression among the different isolates, were also determined to reinforce the proteomic results (data not shown).

### Siderophore production

Siderophore production by the three isolates under various iron concentrations was measured using the Chrome Azurol S (CAS) assay [23]. Briefly, bacterial supernatants were filtered using 0.22 µM filters to remove cells. 500 μL of cell-free supernatant was mixed with 500 μL CAS solution (150 μM CAS, 15 μM FeCl_3_, 0.5 M Piperazine, 1.2 mM HDTMA) (Sigma) and 10 µL shuttle solution (0.2 M 5-Sulfosalicylic acid) (Sigma) and incubated for 5 hours at room temperature. Reference samples of bacterial growth medium containing the relevant concentration of iron were also assayed. Absorbance was measured at 630 nm using MM9 alone as a blank. The percentage siderophore units were calculated as [(Ar - As)/Ar] × 100 where *Ar* is the absorbance of the reference and As is the absorbance of the sample. 

## Results

### IST4113 and IST4134 exhibit a higher capacity for epithelial cell invasion and disruption of epithelial monolayer integrity compared with isolate IST439

**Figure 1:**
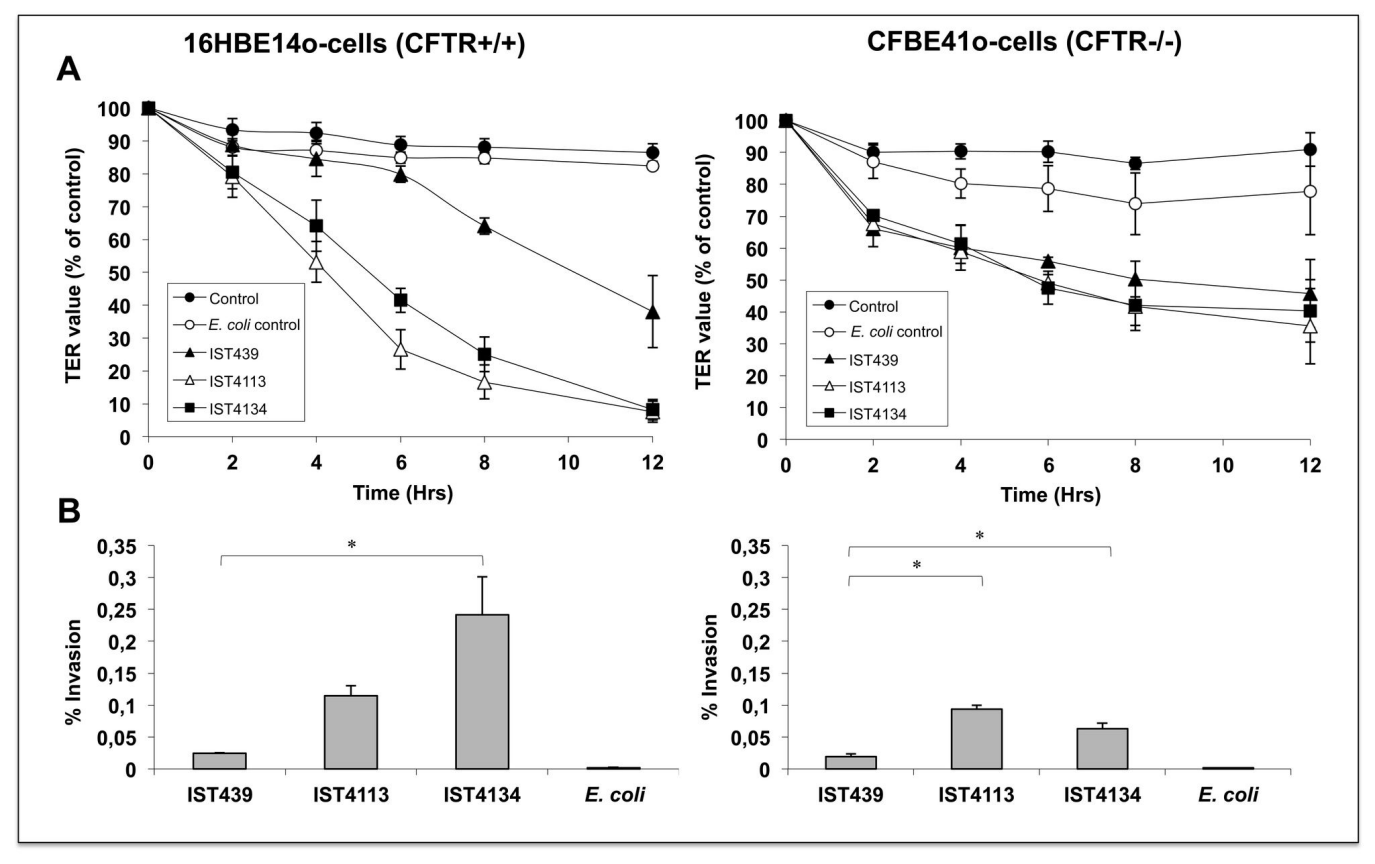
Measure of transepithelial resistance and epithelial cell invasion for *B*. ***cenocepacia* clonal isolates**. (A) Effect of *B*. *cenocepacia* clonal variants IST439, IST4113 and IST4134 on tight junction integrity of 16HBE14o- (CFTR positive) and CFBE41o- (CFTR negative) cells. (B) Invasion potential of the three *B*. *cenocepacia* clonal variants into 16HBE14o- and CFBE41o- epithelial cells. Data represents the mean of 3 individual experiments and error bars represent the standard error of the mean. * P<0.01.

To compare the virulence potential of the three *B. cenocepacia* clonal isolates retrieved during chronic infection examined in this study, polarized epithelial monolayers were separately exposed to isolates IST439, IST4113 and IST4134, and epithelial integrity was analysed. *B. cenocepacia* IST439 was the first isolate recovered from the patient and is believed to have initiated the infection, while IST4134 was recovered 42 months after the isolation date of IST439 and 9 months after IST4113, immediately before the patient’s death with cepacia syndrome and following progressive deterioration of pulmonary function [8,13,24]. Isolate IST439 significantly reduced the transepithelial resistance (TER) of 16HBE14o- (CFTR expressing) monolayers after 8 hours (30%), whereas IST4113 and IST4134 both had a similar impact on TER values as early as 4 hours (Figure 1A). In addition, both IST4113 and IST4134 continued to decrease TER values in 16HBE14o- cells to 10% of controls (cells with no bacteria and with *Escherichia coli* added) over 12 hours (P ≤ 0.001) (Figure 1A). In the independent CFBE41o- epithelial monolayers that do not express functional CFTR, all three isolates rapidly disrupted tight junction integrity to 70% of controls in the first 2 hours with no significant difference between isolates (Figure 1A). Furthermore, there was a more rapid drop in TER in these epithelial monolayers compared to the independently derived 16HBE14o- cells which express functional CFTR upon initial exposure to the bacterial strains (Figure 1A).

 Remarkably, the results on epithelial invasion also follow the same trend: the later isolates are significantly more invasive than IST439 using either CFTR-positive or CFTR-negative cells, but their virulence potential varies with the type of epithelial cells studied (Figure 1B). While IST4134 achieves a percentage of cellular invasion that is twice that of IST4113 in 16HBE14o- (CFTR expressing) cells, IST4113 is more invasive of CFBE41o- epithelial cells than IST4134 (Figure 1B). Interestingly, it should be noted that CFBE41o- cells, which carry the CF mutation, are significantly more resistant to invasion by IST4134 (uptake of this isolate is reduced by more than half), while the levels of invasion by the first two isolates are comparable for both CFTR-negative and CFTR-positive cells (Figure 1B). 

### Differential protein expression profile of IST4134 compared to IST439

Using a 2-D DIGE-based quantitative proteomic approach, it was possible to identify major functional groups and cellular functions that are altered in isolate IST4134 compared with IST439. This analysis allowed the separation of around 740 and 850 protein spots in the gels prepared from cytoplasmic and membrane-associated protein fractions, respectively, within a pI range spanning from pH 3 to approximately pH 9, of which 281 protein spots were positively identified by mass spectrometry (Table S1). GRAVY values (grand average of hydropathicity index) were obtained using the software program ProtParam tool [25,26]. Positive GRAVY values are recognised as valid indicators of hydrophobicity and of membrane involvement [27–29]. In total, 16 (20%) of the 80 proteins identified in the membrane-associated protein fraction had positive GRAVY values ranging from +0.273 to +0.006. Together, the GRAVY index, the transmembrane mapping and the subcellular localisation predictions [13] revealed an enrichment of proteins with a hydrophobic nature in the membrane-associated fraction.

 Overall, 72 protein spots were found to have a different content (fold-change cut-off: 1.5-fold increase or 0.67-fold decrease; P ≤ 0.05, ANOVA) between isolates IST4134 and IST439. Sixty-one of these protein spots, corresponding to 52 different proteins, overlapped the 89-protein dataset previously reported for the comparison between IST4113 and IST439 [13]. All 52 proteins were altered in the same direction in both isolates (Table 1 and Table S1). This subset of proteins will be given a particular emphasis in light of its potential involvement in the enhanced virulence potential and persistence of the last two isolates.

**Table 1:** Relative fold-change of normalised protein spot intensities in 2-D DIGE gels corresponding to the cytoplasmic (spots 1-200) and to the membrane-associated (spots 201-281) enriched fractions of *B. cenocepacia* clonal isolates IST4113 [13] and IST4134 (this work) compared with IST439, and IST4134 compared with IST4113, using a fold-change cut-off of 1.5 (increased content) or 0.67 (decreased content).

						Fold-Change				
Spot No.	UniProtKB best hit		J2315 homolog gene	Gene Name	Protein Function	4113 439	4134 439	4134 ^*^ 4113	ANOVA^a^	
		*Nucleotide metabolism*								
3	Q1BXD4		*BCAL1262*	*carB*	Carbamoyl phosphate synthase large subunit	2.2	1.6	0.7		0.008
33	A2VSP0		*BCAL3261*	*purM*	Phosphoribosylaminoimidazole (AIR) synthetase	2.3	1.5	0.7		<0.001
105	Q39FS0		*BCAL1873*	*purA*	Adenylosuccinate synthetase	0.7	0.7	1.0		0.010
113	Q0BI80		*BCAL3336*	*purH*	Bifunctional purine biosynthesis protein PurH	5.2	2.1	0.4		<0.001
115	Q1BHF2		*BCAL2061*	*guaA*	GMP synthase [glutamine-hydrolyzing]	1.5	1.4	0.9		0.014
121	A4JF47		*BCAL2063*	*guaB*	Inosine-5‘-monophosphate dehydrogenase	3.2	2.1	0.7		0.007
		*Protein folding*								
2	O68191		*BCAL3270*	*dnaK*	Chaperone protein DnaK	2.0	2.2	1.1		0.011
154	O68191		*BCAL3270*	*dnaK*	Chaperone protein DnaK	2.1	1.4	0.7		0.012
8	A4JES5		*BCAL1919*	*clpB*	ClpB heat-shock protein	6.0	2.7	0.4		<0.001
159	A4JES5		*BCAL1919*	*clpB*	ClpB heat-shock protein	5.4	2.8	0.5		<0.001
29	A9AJR0		*BCAL1996*	*clpP*	ATP-dependent Clp protease proteolytic subunit	0.7	0.9	1.2		<0.001
130	Q9ZFE0		*BCAL3146*	*groEL*	60 kDa chaperonin 1	1.7	1.2	0.7		0.004
176	Q0BEF5		*BCAL1997*	*tig*	Trigger factor	2.6	1.9	0.7		<0.001
233	Q1BH74		*BCAL1985*		Putative exported isomerase	1.5	1.5	1.0		0.091
		*Transcription*								
258	Q39KI0		*BCAL0221*	*nusG*	Transcription antitermination protein nusG	0.6	0.6	1.0		0.041
		*Transcriptional regulation*								
248	Q0BAX7		BCAL0499		Two-component regulatory system, response regulator protein	0.9	0.7	0.8		0.019
		*Translation*								
4	Q1BZ70		*BCAL3373*	*leuS*	Leucine-tRNA ligase	3.4	2.5	0.7		<0.001
7	A4JDU9		*BCAL1486*	*pheT*	Phenylalanine-tRNA ligase beta subunit	3.6	1.9	0.5		<0.001
17	B2JF06		*BCAL2950*	*rpsA*	30S ribosomal protein S1	2.7	2.2	0.8		<0.001
18	B2JF06		*BCAL2950*	*rpsA*	30S ribosomal protein S1	2.3	2.0	0.8		0.003
177	B2JF06		*BCAL2950*	*rpsA*	30S ribosomal protein S1	2.4	2.5	1.1		0.032
22	Q123F6		*BCAL0232*	*tuf*	Elongation factor Tu	2.9	1.8	0.6		<0.001
178	Q1BX19		*BCAL1416*	*alaS*	Alanine-tRNA ligase	2.9	2.0	0.7		<0.001
181	A2VV06		*BCAL0679*	*argS*	Arginine-tRNA ligase	2.9	1.7	0.6		<0.001
183	Q1BSL2		*BCAL0484*	*gatA*	Glutamyl-tRNA (Gln) amidotransferase subunit A	4.1	2.2	0.5		<0.001
186	Q1BRU5		*BCAL0231*	*fusA*	Elongation factor G1	2.5	1.7	0.7		<0.001
187	A4JH32		*BCAL2724*	*ileS*	Isoleucine-tRNA ligase	2.5	1.8	0.7		<0.001
190	Q0BE16		*BCAL2090*	*tsf*	Elongation factor Ts	2.3	1.4	0.6		<0.001
256	Q1BT08		*BCAL0630*		Putative uncharacterized protein	0.5	0.6	1.4		0.017
		*Amino acid metabolism*								
16	Q1BHV4		*BCAL2213*		Oligopeptidase A	2.5	1.4	0.6		0.009
36	Q39BX5		*BCAL0496*	*argB*	Acetylglutamate kinase	1.6	1.1	0.7		0.002
37	Q39BX5		*BCAL0496*	*argB*	Acetylglutamate kinase	2.4	1.3	0.6		<0.001
52	A2VXX6		*BCAL1874*	*hisZ*	ATP phosphoribosyltransferase regulatory subunit	2.2	1.7	0.8		<0.001
90	Q1BGN7		*BCAL1796*		Putative saccharopine dehydrogenase	0.7	0.8	1.1		0.001
96	Q1BGN7		*BCAL1796*		Putative saccharopine dehydrogenase	0.7	0.8	1.1		0.027
143	A0AXU0		*BCAM0512*		Putative aminotransferase	2.5	1.6	0.6		<0.001
153	A2VTH5		*BCAL0312*	*hisD*	Histidinol dehydrogenase	1.5	1.2	0.8		0.005
243	Q1BHV9		*BCAL2221*		Putative prolyl oligopeptidase	1.2	2.7	2.3		0.076
255	A2VU73		*BCAL0147*	*metF*	5,10-methylenetetrahydrofolate reductase	0.5	0.5	1.0		0.046
270	A2VSL1		*BCAL3197*	*glyA1*	Serine hydroxymethyltransferase	0.7	0.9	1.3		0.047
277	A4JGW1		*BCAL2641*		Putative ornithine decarboxylase	0.4	0.4	1.0		0.016
		*Cell envelope biogenesis*								
15	A2W339		*BCAM2430*		Putative biotin carboxylase	2.0	1.4	0.7		0.025
35	A2VT63		*BCAL3460*	*ddl*	D-alanine-D-alanine ligase	2.0	1.4	0.7		<0.001
44	A2VT78		*BCAL0409*	*paaF*	Putative phenylacetic acid degradation enoyl-CoA hydratase PaaF	0.7	0.8	1.1		0.010
149	A2VVY4		*BCAL2783*		Putative cyclopropane-fatty-acyl-phospholipid synthase	3.1	1.5	0.5		<0.001
156	A2VWW0		*BCAL2284*	*acoE*	Acetyl-CoA synthetase 1	1.9	1.3	0.7		<0.001
167	A2VT29		*BCAL3420*	*accB*	Acetyl-CoA carboxylase biotin carboxyl carrier protein subunit	0.6	0.8	1.3		0.008
198	Q13XC7		*BCAL2080*	*fabZ*	(3R)-hydroxymyristoyl-(acyl carrier protein) dehydratase	0.7	0.9	1.3		0.044
201	A2VXD7		*BCAL2083*		Outer membrane protein assembly factor YaeT	4.7	2.0	0.4		0.018
242	A2VXD7		*BCAL2083*		Outer membrane protein assembly factor YaeT	1.2	1.7	1.4		0.075
212	A2VQ60		*BCAL1829*		Putative outer membrane protein	1.6	2.0	1.3		0.084
267	B1JX22		*BCAL3113*	*manB*	Phosphomannomutase	0.7	0.6	0.9		0.057
269	A4JCW7		*BCAL1071*		NAD-dependent epimerase/dehydratase	0.6	0.5	0.8		0.017
		*Energy metabolism*								
9	Q59097		*BCAL2209*	*aceE*	Pyruvate dehydrogenase E1 component	3.5	2.0	0.6		<0.001
155	Q59097		*BCAL2209*	*aceE*	Pyruvate dehydrogenase E1 component	3.4	1.9	0.6		<0.001
160	Q59097		*BCAL2209*	*aceE*	Pyruvate dehydrogenase E1 component	3.4	1.8	0.5		<0.001
10	Q0BAG3		*BCAM0961*	*acnA*	Aconitate hydratase	3.0	1.9	0.6		0.008
13	A2W536		*BCAL3389*	*tktA*	Transketolase 1	1.5	1.3	0.8		0.009
254	A2W536		*BCAL3389*	*tktA*	Transketolase 1	0.5	0.5	1.0		0.014
38	Q1BY10		*BCAL2934*	*etfA*	Electron transfer flavoprotein, alpha subunit	1.6	1.3	0.8		0.008
257	Q1BY10		*BCAL2934*	*etfA*	Electron transfer flavoprotein, alpha subunit	0.5	0.7	1.2		0.002
47	A2VSX6		*BCAL3366*	*eda*	KHG/KDPG aldolase	1.5	1.4	0.9		0.002
81	Q0B5L9		*BCAM0042*		Putative aldo/keto reductase	2.3	1.4	0.6		<0.001
87	A2VSZ8		*BCAL3388*	*gapA*	Glyceraldehyde-3-phosphate dehydrogenase 1	0.7	0.8	1.2		0.094
92	A2VVT0		*BCAL2839*	*cbbA*	Fructose-bisphosphate aldolase	2.1	1.3	0.6		<0.001
108	A2WDT7		*BCAM0972*	*gltA*	Citrate synthase	2.3	1.3	0.6		0.001
109	B1YP46		*BCAL1517*	*odhL*	Dihydrolipoyl dehydrogenase	3.0	2.4	0.8		<0.001
189	B1YP46		*BCAL1517*	*odhL*	Dihydrolipoyl dehydrogenase	1.7	1.4	0.8		<0.001
112	A2W309		*BCAM2468*		Putative aldehyde dehydrogenase family protein	3.4	2.1	0.6		<0.001
132	A2VWQ9		*BCAL2338*		NADH-quinone oxidoreductase	3.6	2.4	0.6		0.002
147	A4JFY5		*BCAL2179*	*eno*	Enolase	0.7	0.9	1.2		0.017
168	Q1BJZ1		*BCAM2821*	*glcB*	Malate synthase G	2.6	1.3	0.5		<0.001
170	Q1BNJ4		*BCAM1581*	*pckG*	Phosphoenolpyruvate carboxykinase	3.8	2.2	0.6		<0.001
171	Q1BNJ4		*BCAM1581*	*pckG*	Phosphoenolpyruvate carboxykinase	3.9	2.2	0.6		<0.001
204	A2VZR7		*BCAM0969*	*sdhA*	Succinate dehydrogenase flavoprotein subunit	1.5	1.8	1.2		0.099
205	A2VZR7		*BCAM0969*	*sdhA*	Succinate dehydrogenase flavoprotein subunit	1.6	1.7	1.1		0.040
238	A2VZR7		*BCAM0969*	*sdhA*	Succinate dehydrogenase flavoprotein subunit	1.1	0.5	0.5		0.025
215	A2W2F4		*BCAM2710*		Putative acetyl-CoA synthetase	4.7	1.5	0.3		0.048
216	B1K5B2		*BCAM1537*		Putative dehydrogenase, zinc-binding subunit	1.4	1.5	1.1		0.081
227	A2VTZ2		*BCAL0034*	*atpA*	ATP synthase subunit alpha	1.8	1.8	1.0		0.014
229	Q1BNJ5		*BCAM1588*		Isocitrate lyase	1.5	1.4	0.9		0.023
236	A2WG89		*BCAM2323*		Putative N-methylproline demethylase	1.3	2.2	1.7		0.086
253	Q1BRR9		*BCAL0205*		NADP-dependent malic enzyme	0.3	0.5	1.7		0.065
261	A2WBM9		*BCAL2908*	*fumC*	Fumarate hydratase class II	0.6	0.5	0.8		0.012
262	Q1BNF8		*BCAM1542*		Putative aldehyde dehydrogenase	0.6	0.6	1.0		0.052
272	Q1BJI6		*BCAM2702*	*prpC*	2-methylcitrate synthase	1.3	1.7	1.7		0.055
276	A9AIN1		*BCAL2074*	*ppsA*	Phosphoenolpyruvate synthase	0.5	0.5	0.5		0.016
		*Iron transport*								
68	B1YXQ3		*BCAM0026*		Putative siderophore-interacting protein	3.2	1.9	0.6		0.033
202	A2W3M3		*BCAM2224*		Putative pyochelin receptor protein FptA	1.8	1.6	0.9		0.016
203	Q1BM21		*BCAM0948*		TonB-dependent receptor	1.8	1.5	0.8		0.017
240	Q1BWE3		*BCAL1691*	*orbC*	Putative iron transport-related ATP-binding protein	1.0	1.5	1.5		0.010
		*Intracellular trafficking and secretion*								
232	A2VW75		*BCAL2669*		Hypothetical protein	1.7	1.7	1.0		0.011
		*Secondary metabolites biosynthesis*								
34	A2W219		BCAM0023	adc	Probable acetoacetate decarboxylase	2.3	1.4	0.6		<0.001
		*Coenzyme metabolism*								
20	A4JIQ4		*BCAL0509*	*metK*	S-adenosylmethionine synthetase	0.7	0.9	1.3		0.001
69	A0K8R4		*BCAL2212*	*folD*	Bifunctional protein FolD	1.6	1.4	0.9		0.026
64	A4JKF8		*BCAM0022*		Putative D-beta-hydroxybutyrate dehydrogenase	2.1	1.4	0.7		<0.001
		*Replication*								
220	Q1BLY0		*BCAM0904*	*polA*	DNA polymerase I	1.1	1.8	1.6		0.078
		*Adaptation to atypical conditions*								
75	A2VVV1		*BCAL2816*		S-formylglutathione hydrolase	1.6	1.4	0.8		0.038
25	A2VT33		*BCAL2013*		AhpC/TSA family protein	0.7	1.0	1.4		0.002
252	A2VXR9		*BCAL1937*		Putative phosphorous metabolism-related protein	0.5	0.5	1.0		0.015
		*Transport of small molecules*								
213	Q1BGR3		*BCAL1823*	*potG*	Putrescine ABC transporter ATP-binding protein	1.2	1.8	1.5		0.077
231	A2W3K2		*BCAM2247*	*livF*	Putative amino acid ABC transporter ATP-binding protein	1.5	1.4	0.9		0.019
235	A9AH88				ABC transporter related (Polar amino acid transport)	1.6	2.5	1.6		0.046
263	Q1BIT4		*BCAS0242*		Hypothetical protein	0.6	0.6	1.0		0.041
		*Adhesion*								
266	Q1BKJ6		*BCAM0184*	*bclB*	Lectin (Fucose-binding lectin II)	0.6	0.6	1.0		0.056
		*Unknown*								
128	Q1BJ23		*BCAS0293*	*aidA*	Nematocidal protein AidA	3.0	3.2	1.1		<0.001
131	B1KBA2		*BCAS0292*		Hypothetical protein	0.7	0.8	1.1		0.017
207	B1KBA2		*BCAS0292*		Hypothetical protein	0.8	0.7	0.9		0.043
250	A2VS76		*BCAL3052*		Hypothetical protein	0.8	0.7	0.9		0.015

For each protein, the Genebank accession number of the best-hit protein identified by MS and the respective J2315 homolog gene are indicated.

^a^ Values were calculated as the average data from two independent experiments (six replicates of each sample) and data were filtered to retain spots with ANOVA P-value lower than 0.05. In some specific cases, results with ANOVA P-value up to 0.05 are shown.

*vs.* IST439 and IST4134 *vs.* IST439. ^*^ Indirect determination based on the analysis of IST4113

The 30S ribosomal protein S2 (BCAL2091, spot 219) was used as a reference spot control, since its quantity remained constant across all replicates and strains. Individual spot volumes were automatically normalised against the total volume for a given gel using Progenesis Samespots software.

**Figure 2:**
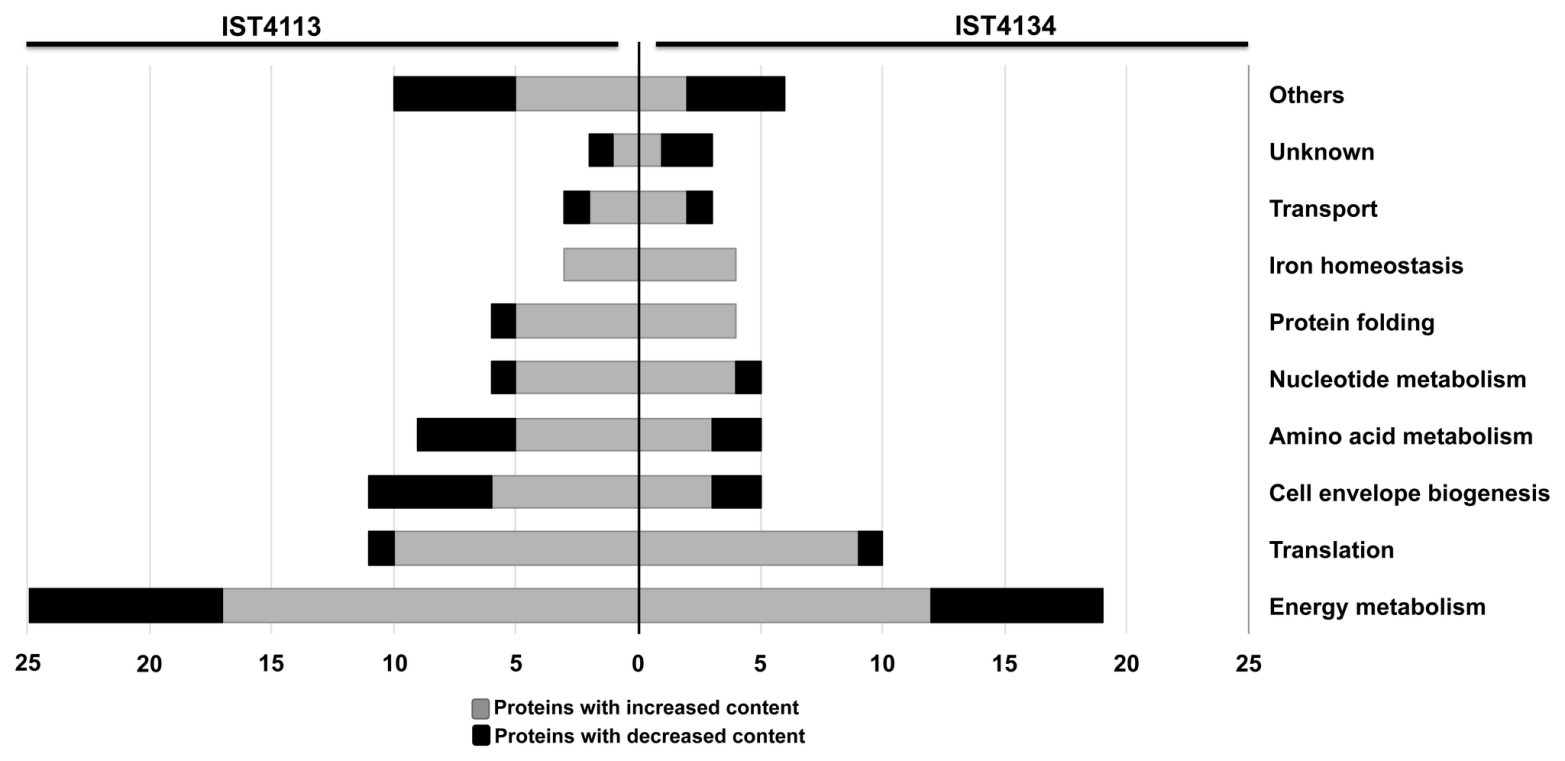
Clustering of the proteins differently expressed in *B*. ***cenocepacia* clonal variants, based on biological function**. Number of proteins differently expressed in isolates IST4113 [13] or IST4134 (this work), compared to IST439, grouped by functional categories (as detailed in Table 1), based on the information available in the *Burkholderia* Genome database and in the KEGG Pathways Database. All categories that include less than 2 proteins were grouped together as “Others”; this category includes proteins involved in “Coenzyme metabolism”, “Intracellular trafficking and secretion”, “Secondary metabolites biosynthesis”, “Transcription” Transcriptional regulation”, “Replication”, “Adaptation to atypical conditions” and “Adhesion”.

 Best-hit homologs to *B. cenocepacia* J2315 genes were found for all proteins identified in the comparison between IST4134 and IST439, with the exception of the ABC transporter-related protein A9AH88, with 97% of identity to the L-glutamate ABC transporter ATP-binding protein encoded by gene Bcen_0189 from *B. cenocepacia* AU1054, a strain that was recovered from the blood of a CF patient [30] (Table 1). Proteins that were suggested to be differently expressed in both IST4113 and IST4134, compared with IST439, were clustered into functional categories (Table 1 and Figure 2). Considering only the analysis of IST4134 *vs.* IST439, the most prominent category is “Energy metabolism” (n = 19 proteins; Table 1 and Figure 2), similarly to what was previously described for IST4113 [13]. The second main category is “Translation”, including a total of 10 proteins (9 of which are over-expressed) with altered content in IST4134. “Cell envelope biogenesis”, “Amino acid metabolism” and “Nucleotide metabolism” follow, each comprising 5 proteins with altered content in IST4134. The categories “Protein folding” and “Iron transport” are noteworthy since all proteins that were considered altered in IST4134 are over-expressed compared to IST439. A more detailed description of the main results of this quantitative proteomic analysis can be found below.

### Proteomic differences between IST4113 and IST4134 detected indirectly

 Based on the fold-change ratios determined for each protein spot in the comparison of IST4134 *vs.* IST439 [13] and IST4134 *vs.* IST439 (Table 1), we were able to determine indirect proteomic differences between IST4134 and IST4113, with possible relevance to understand disease progression. Considering a cut-off of 1.5-fold increase or 0.67-fold decrease in IST4134 (*P* < 0.05) 40 proteins are altered significantly from IST4113 to IST4134 (Table 1). Considering a more significant fold-change cut-off (2-fold increase or 0.5-fold decrease), 9 proteins were singled-out, including the heat shock chaperone ClpB, the *C. elegans* virulence factor GatA, the nucleotide metabolism protein PurH, and 2 proteins involved in cell envelope biogenesis, BCAL2783 (lipid synthesis) and the assembly factor YaeT – all with reduced content in IST4134 *vs.* IST4113.

### The content of proteins involved in purine and pyrimidine synthesis is higher in isolate IST4134 compared to IST439

In general, proteins involved in the *de novo* purine synthesis pathway, namely PurM, PurH and GuaB, exhibit a higher content in the later isolates than in IST439 [13] (Table 1). The exception is PurA, an adenylosuccinate synthetase that catalyses the first step of the conversion of inosinate into adenosine monophosphate (AMP) in the aforementioned pathway. This protein is less abundant in IST4134 than in IST439 (Table 1), as previously described for IST4113 [13]. The higher content of proteins involved in the alternative step that converts inosinate into guanosine monophosphate (GMP), in particular GuaB, in both IST4113 and IST4134, reinforces the previously suggested preference for the synthesis of GTP *versus* AMP in both isolates, compared to IST439 [13]. The content of proteins involved in pyrimidine synthesis is also increased in both IST4113 and IST4134 (Table 1), in particular CarB, one of the two subunits of the first enzyme involved in this anabolic pathway [31]. 

### Proteins involved in translation and protein folding have a higher content in IST4134 compared to IST439

A total of 9 proteins involved in translation processes were found to have a higher content in isolate IST4134, compared to IST439, including components of the ribosome (RpsA, 3 spots, with fold-changes ranging from 2.0 to 2.5), elongation factors (Tuf and FusA), and tRNA synthetases (LeuS, PheT, AlaS, ArgS, GatA and IleS) (Table 1). The content of all these proteins is also altered in the highly antimicrobial resistant isolate IST4113, compared to IST439 [13] (Table 1). Overall, the results indicate that translation is more active in IST4134 than in the isolate that initiated the infection, as suggested previously for IST4113 based on transcriptomic and proteomic data [12,13]. 

 The content of several proteins belonging to the category “Protein folding” is higher in IST4134 than in IST439, including the chaperone protein DnaK, the heat shock protein ClpB, and the trigger factor Tig (Table 1). DnaK was described in the Gram-negative human pathogen *Brucella suis* as playing an essential role in the protein repair system that protects the bacteria from the environment in the macrophage phagosome [32]. Of the 72 protein spots whose content is altered in isolate IST4134 compared to IST439, the most significantly altered protein is ClpB, identified in two different spots (Table 1). 

### The content of proteins involved in cell envelope biogenesis is altered in isolate IST4134 compared to IST439

Proteins involved in lipopolysaccharide (LPS) biosynthesis, namely the phosphomannomutase ManB and the NAD-dependent epimerase, were found to have a lower content in the last isolate compared to IST439, as previously described for IST4113 [13] (Table 1). ManB is involved in lipid A synthesis while the NAD-dependent epimerase protein is mainly involved in the synthesis of core oligosaccharide and O-antigen, which are components of the lipopolysaccharide molecule. Both forms of the outer membrane protein assembly factor YaeT, which is involved in protein extrusion to the outer membrane maintaining a homeostatic LPS to protein ratio [33,34], have a higher content in isolate IST4134 than in IST439. 

### Late isolates exhibit an increased content of iron uptake proteins and are able to capture iron more efficiently from the environment

**Figure 3:**
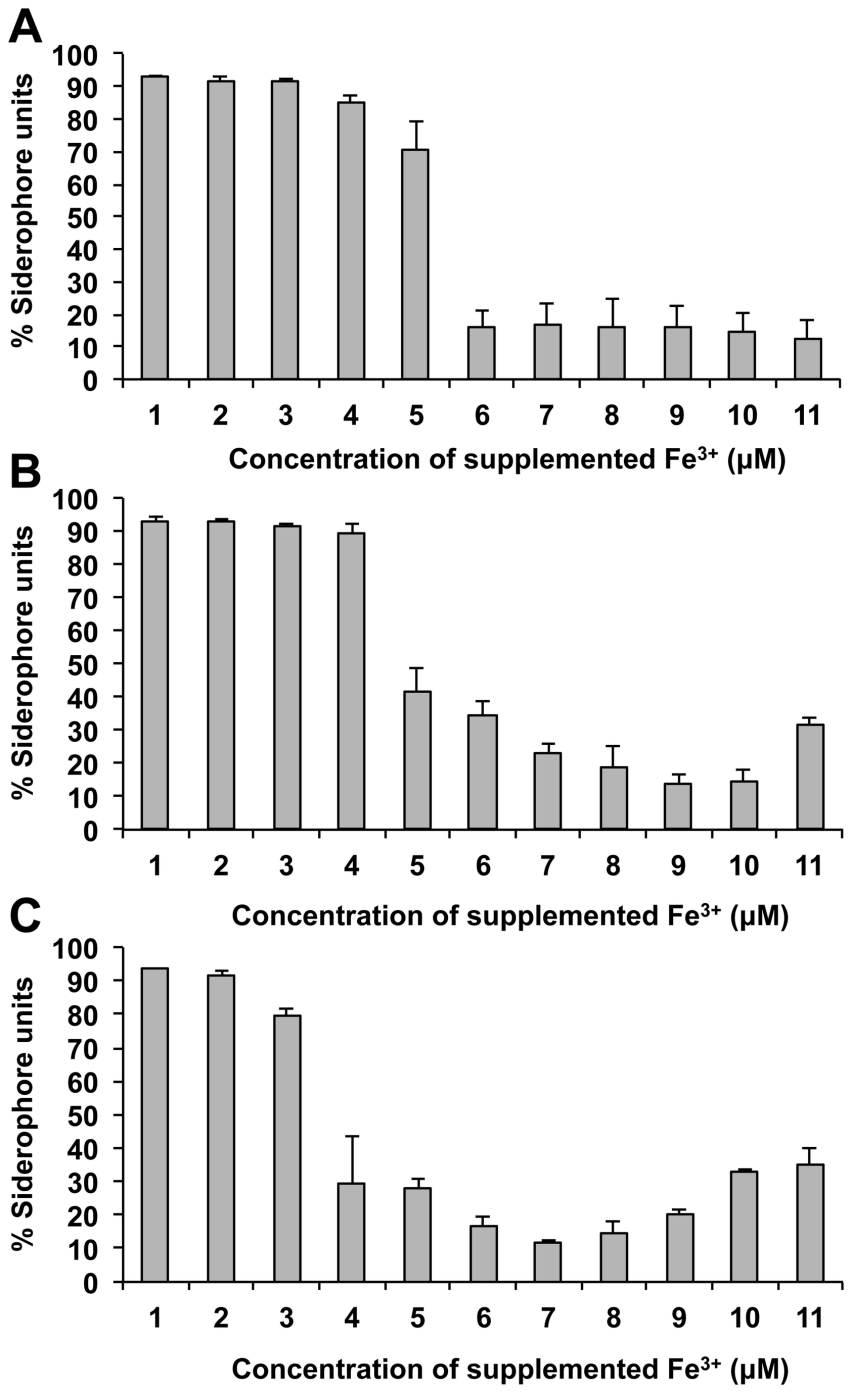
Siderophore production measured by CAS assay for the three *B*. ***cenocepacia* clonal variants**. Siderophore production was measured for isolates IST439 (A), IST4113 (B) and IST4134 (C) under different iron concentrations (1 to 11 μM of Fe^3+^) added to minimal M9 media.

Four proteins involved in iron uptake are more abundant in IST4134, compared to IST439 (Table 1). Three of these are also over-expressed in IST4113 compared to IST439: putative siderophore-interacting protein, putative pyochelin receptor protein FptA and TonB-dependent receptor [13]. OrbC, a putative iron transport-related ATP-binding protein whose gene expression is up-regulated by OrbS under iron deprivation conditions [35], was only over-expressed in isolate IST4134, compared to IST439 (Table 1). These results led us to examine the production of siderophores by the three clonal variants, using the CAS assay with different iron concentrations (Figure 3). Significant levels of siderophores were secreted by the three *B. cenocepacia* isolates within two hours of exposure to an iron-depleted environment, demonstrating the ability of this species to rapidly adjust to low iron availability (Figure 3). The results obtained show that IST4113 and IST4134 are more tolerant to low iron concentrations than IST439. The latter produces significantly higher levels of siderophores below 6 µM of Fe^3+^ (Figure 3A), while isolates IST4113 and IST4134 only up-regulate siderophore production below 5 µM or 4 µM of Fe^3+^, respectively (Figure 3B, C). 

### Alteration of proteins involved in metabolic processes

Energy and central intermediary metabolism functions are strikingly overrepresented among proteins whose content is altered in IST4134, compared to IST439 (Table 1, Figure 2). This is the case of several proteins of the tricarboxylic acid (TCA) cycle (OdhL, AcnA, SdhA and FumC) and other pathways like glycolysis/gluconeogenesis (Putative aldehyde dehydrogenase family protein), pyruvate metabolism (PpsA, AceE, OdhL and PckG), and oxidative phosphorylation (NADH-quinone oxidoreductase, SdhA and AtpA) (Table 1). In total, 19 of the 23 protein spots that are differently abundant in isolate IST4134 compared to IST439 are also altered in IST4113 [13] (Table 1). However, a number of proteins with functions in cell metabolism that were altered in IST4113 did not have their expression altered in IST4134, or exhibited a less significant variation. 

## Discussion

In this study we assessed the virulence potential of three *B. cenocepacia* clonal isolates involved in a 3.5-year long chronic infection of a CF patient, from the onset of infection until death with the cepacia syndrome, and explored a quantitative proteomic analysis to uncover key mechanisms of pathogenesis during progressive CF lung disease. Although genome-wide expression analyses at the transcript level have been previously applied to study *B. cenocepacia* virulence in a CF context [12,30], mRNA levels do not always correlate directly with protein levels. In spite of its lower coverage, quantitative proteomics is an invaluable tool to supply complementary quantitative and functional data, allowing a more accurate understanding of the biological functions involved.

 The two isolates recovered during late-stage CF lung disease (IST4113 followed 9 months later by IST4134, immediately before the patient’s death) were shown to be significantly more virulent than the clonal isolate believed to have initiated the infection, IST439. In fact, all three isolates were able to disrupt the integrity of 16HBE14o- (CFTR expressing) monolayers, however the effect of IST4113 and IST4134 was similarly swifter (4 hours *vs.* 8 hours for IST439), suggesting that genetic adaptation of the initial isolate during the course of the chronic infection led to an enhanced ability of the last isolates to gain access to underlying tissue. Although it could be expected that the isolate retrieved immediately before the patient’s death (IST4134) would be more effective than IST4113, this was not the case. It is likely that the effect of IST4113 was maximal and could not be further augmented. Interestingly, there were no differences between isolates in CFBE41o- (CFTR negative) cells. The effect on monolayer integrity was also more rapid, which may be significant *in vivo*. These observations might be attributed to the tight junction regulatory protein Zo-1. We have previously shown that Zo-1 in CFBE41o- cells is more susceptible to disruption by Bcc infection than in 16HBE14o- cells [36], so it is probable that the early isolate had an optimal impact on the tight junctions of the CFBE41o- cells which was not further disrupted by the later isolates.

 The results on invasion potential are comparable, but more dependent on the type of epithelial cells used. The more resistant phenotype of cells lacking a functional CFTR to IST4134 might be correlated with our previous findings that *B. cenocepacia* strain J2315 invasion was significantly reduced in CFBE41o- cells relative to 16HBE14o- cells [36]. In fact, CFTR-mediated cellular invasion has been described for some pathogens including *P. aeruginosa* [37], and although this has not been reported for *B. cenocepacia*, it may account for the diminished levels of invasion of CFBE 41o- cells. In addition, the absence of the CFTR receptor will change many aspects of cellular physiology, which could potentially alter susceptibility to invasion. Finally, it should also be noted that these are not paired bronchial epithelial cell lines and so differences other than the CFTR receptor will further confound comparisons of data derived from these two cell lines. The loss of epithelial monolayer integrity in the presence of the isolates and their ability to invade epithelial cells are two distinct bacterial host interactions, but both potentially facilitate effective and persistent colonisation of the lung tissue. Moreover, a correlation between *in vitro* invasion of epithelial cells by *B. cenocepacia* and *in vivo* virulence in a mouse model has previously been demonstrated [38] and suggests that increased epithelial invasiveness by the later isolates may have implications for *in vivo* infection.

 The proteomes of IST4134 and IST439 were compared quantitatively and integrated with a previous report focusing on the analysis of the highly antimicrobial resistant isolate IST4113 *vs.* IST439 [13]. The key aim was to identify groups of proteins and/or functions that were similarly altered in IST4113 and IST4134 and could therefore be associated with their increased potential for tissue invasion compared with IST439. The proteins and functions where the proteomic profiles (and therefore the adaptive evolution) of IST4113 and IST4134 diverged were also a point of interest given its relevance to the understanding of late-stage progression of the disease. 

 There is a marked similarity between the alterations in both IST4113 and IST4134 compared to IST439, suggestive of a convergence in the adaptive evolution of both isolates in the lung concomitant with disease progression (IST4113 and IST4134 were collected within 9 months of each other). Of particular interest are the following pathways and processes: 1) up-regulation of protein translation and folding, 2) up-regulation of purine and pyrimidine biosynthesis, 3) LPS biosynthesis 4) up-regulation of iron uptake and scavenging, and 5) metabolic adaptation. Both IST4113 and IST4134 are more resistant to antibiotics targeting protein translation [8], which is in agreement with the increased content of proteins involved in translation, although more marked in the highly resistant IST4113 (Table 1). The same is observed for proteins involved in protein folding, stabilisation or in prevention of protein aggregation, which is likely to contribute to the adaptive response of the colonising bacteria to the stressing conditions of the CF airways [39–41]. The increase in nucleotide biosynthetic activity is also suggestive of a better performance of repair processes in the adapted isolates, which can be induced by host defences or antimicrobial therapy [42]. Importantly, genes encoding enzymes of the purine and pyrimidine biosynthesis pathways were recently shown to be essential for *B. cenocepacia* H111 virulence in multiple non-mammalian host models of infection [43]. Survival and growth of bacteria in the human bloodstream is also dependent on *de novo* purine and pyrimidine biosynthetic pathways [44]. Overall, these indications propose an association between the increased expression of purine and pyrimidine biosynthetic pathways in IST4113 and IST4134 and their greater capacity for tissue invasion.

 The proteomes of both IST4113 and IST4134 reflect a decreased biosynthesis of LPS or the synthesis of a modified molecule compared to IST439. Remarkably, recent studies on the evolution of *B. cenocepacia* and *B. dolosa* species have unveiled a mechanism of convergent evolution involving the gene cluster responsible for synthesising the O-antigen as well as other LPS-related genes, both *in vivo* and *in vitro*, highlighting a probable role of altered LPS synthesis in adaptation and persistence [9,18]. The proteomic results also indicate that the late-stage clonal isolates are able to capture iron more efficiently from the environment [45]. The proteomic analysis was corroborated by further results showing that the later isolates are better adapted to low iron conditions, as demonstrated by the levels of siderophores secreted under different iron availability conditions (Figure 3B, C). This adaptation evidenced by IST4113 and IST4134 is a relevant mechanism that might contribute to the increased survival and persistence of both isolates in the CF lung. Interestingly, a recent study of experimental evolution of *B. cenocepacia* in biofilms identified convergent mutations in pathways involved in iron scavenging, suggesting that this adaptive mechanism might be favoured by the biofilm environment rather than an iron-limited environment [18].

 Remarkably, out of 40 proteins with a significant difference between IST4134 *vs.* IST4113, only 2 had a higher content in IST4134, suggesting a general decrease in expression for all proteins identified, in particular those involved in metabolic pathways, such as amino acid, nucleotide and energy metabolism. Furthermore, there are several proteins in the latter category that are altered in IST4113 but not in IST4134 (Table 1). These results might reflect a mechanism of metabolic reprogramming and optimisation occurring alongside disease progression, where the bacteria continuously adjust their metabolic requirements to the evolving host environment, as previously seen for *B. cenocepacia* [46] and *P. aeruginosa* [47,48]. This metabolic reprogramming that underlies several of the alterations described above is likely an adaptation to the nutritional and microaerobic conditions experienced by the two later isolates in the lung of the CF patient. Recent studies are indicative of lowered oxygen tension in the mucus of CF patients and have shown that *B. cenocepacia* is well adapted to these conditions [49]. This has also been substantiated by reports of parallel evolution of oxygen-dependent regulatory pathways in *B. dolosa* [50]. We have found that 23 proteins that are altered in the two later isolates are also involved in the response of *B. cenocepacia* J2315 to low oxygen availability [49], encompassing protein folding, cell envelope biogenesis and energy metabolism (e.g. fatty acid metabolism; Table S2). These observations highlight the complexity of the CF lung host environment, where adaptive mechanisms become intertwined as the colonising population faces varying selective pressures. Increases in proteins involved in the metabolism of fatty acids have also been previously associated with adaptation to low oxygen in *P. aeruginosa* isolates [48], and are consistent with a previously proposed adaptive strategy involving a reduction of the fatty acid saturation degree in association with severe oxygen depletion in the CF lung [8]. It should be noted that the clinical clonal isolates used in this study were grown under aerobic conditions, and therefore we were not able to identify proteins that are induced specifically under oxygen limitation or anaerobiosis.

 Although isolates IST4134 and IST4113 were found to have greater virulence potential than IST439, only two proteins described as virulence factors in *B. cenocepacia*, AidA and GatA, were altered in the proteomes of the last two isolates (this study and ref [13].). However, even though both proteins were shown to be involved in nematode pathogenicity [43,51], the same has not been demonstrated in other infection models, including *G. mellonella*, mice or rats [11,52]. For this reason, a correlation between the different epithelial invasiveness of these isolates and the content of these virulence factors in the corresponding proteomes cannot be established. The absence of other classical virulence factors might be at least partially explained by the limited coverage of the 2-DE-based proteomic analysis, particularly at the level of cell membrane, membrane-associated and secretory proteins [53]. However, this is in line with the general conclusions of recent studies using genome-wide approaches, in particular based in the screening of mutant libraries to identify virulence determinants [11,54,55]. Furthermore, it is also in agreement with an ongoing analysis of the secretome of the three isolates (manuscript under preparation) aiming to identify secreted proteins with a role in pathogenesis [16,56]. Finally, it is also worth noting that the culture conditions of the bacteria will undoubtedly impact on their proteomic profile and the data may not be reflective of all proteomic changes that occur in the competitive environment of the host. 

 In conclusion, our results reinforce the idea that adaptation to the host environment is essential for the development of chronic infections by *B. cenocepacia* and cannot be dissociated from the rapid deterioration of lung function observed during late-stage disease [11,43,46]. Metabolic pathways are proposed to play a crucial role in the adaptation and persistence of *B. cenocepacia* in the CF lung, in line with previous reports of gene expression alteration during chronic CF infections with both Bcc and *P. aeruginosa* species [46–48]. Future work will focus on the exploitation of the different hypotheses raised throughout this study, namely in the verification of specific mechanisms responsible for the increased virulence potential and ability to persist of late-stage isolates.

## Supporting Information

Table S1 **Proteins showing different abundance in the three *B. cenocepacia* clonal isolates, identified in the cytoplasmic fraction (spots 1-200) and in the membrane-associated (spots 201-281) enriched fraction (results published before in Madeira et al., 2011).** For each protein, the Swiss-Prot/TrEMBL accession number and GRAVY index is indicated (Gasteiger et al., 2005). Protein identification was obtained by mass spectrometry.
(DOCX)

Table S2 **Proteins whose content was found to be increased or decreased in isolates IST4113 and 4134, compared to IST439, and differently expressed, in the same direction, in cells of *B. cenocepacia* J2315 in response to oxygen limitation, using the corresponding microarray dataset published in Sass et al.** (2013)*.
A fold-change cut-off of ±1.5 was used.
(DOCX)
