# Supplementary material for: Proteomic Profiling of Burkholderia cenocepacia Clonal Isolates with Different Virulence Potential Retrieved from a Cystic Fibrosis Patient during Chronic Lung Infection
Source: PLoS One. 2013 Dec 13;8(12):e83065. doi: 10.1371/journal.pone.0083065 (PMC3862766; doi:10.1371/journal.pone.0083065)
Supplement: Table S1 — Proteins showing different abundance in the three B. cenocepacia clonal isolates, identified in the cytoplasmic fraction (spots 1-200) and in the membrane-associated (spots 201-281) enriched fraction (results published before in Madeira et al., 2011). For each protein, the Swiss-Prot/TrEMBL accession number and GRAVY index is indicated (Gasteiger et al., 2005). Protein identification was obtained by mass spectrometry. (DOCX) [file pone.0083065.s001.docx]

**Table S1 –** Proteins showing different abundance in the three *B. cenocepacia* clonal isolates, identified in the cytoplasmic fraction (spots 1-200) and in the membrane-associated (spots 201-281) enriched fraction (results published before in Madeira *et al.*, 2011). For each protein, the Swiss-Prot/TrEMBL accession number and GRAVY index is indicated (Gasteiger *et al.*, 2005). Protein identification was obtained by mass spectrometry.

| Spot (nr) | Name | UniProtKB/ TrEMBL  *best hit* | Mascot score | Nr. of peptides matched | Sequence coverage (%) | GRAVY Index |
| --- | --- | --- | --- | --- | --- | --- |
| 2 | Chaperone protein DnaK | O68191 | 635 | [20](file:///G:\paper-Bcc\PSR\PSR_0173_1_G14.mht) | [30](file:///G:\paper-Bcc\MSRPV\MSRPV_0173_1_G14.mht) | -0.325 |
| 3 | Carbamoyl phosphate synthase large subunit, CarB | Q1BXD4 | 128 | [10](file:///G:\paper-Bcc\PSR\PSR_0173_1_I14.mht) | [12](file:///G:\paper-Bcc\MSRPV\MSRPV_0173_1_I14.mht) | -0.153 |
| 4 | Leucine-tRNA ligase, LeuS | **Q1BZ70** | 141 | [9](file:///G:\paper-Bcc\PSR\PSR_0173_1_K14.mht) | [20](file:///G:\paper-Bcc\MSRPV\MSRPV_0173_1_K14.mht) | -0.283 |
| 7 | Phenylalanine-tRNA ligase beta subunit, PheT | **A4JDU9** | 120 | [4](file:///G:\paper-Bcc\PSR\PSR_0173_3_A14.mht) | [5](file:///G:\paper-Bcc\MSRPV\MSRPV_0173_3_A14.mht) | -0.129 |
| 8 | ClpB heat-shock protein, ClpB | **A4JES5** | [364](file:///G:\paper-Bcc\PSR\PSR_0173_3_C14.mht) | [17](file:///G:\paper-Bcc\PSR\PSR_0173_3_C14.mht) | [24](file:///G:\paper-Bcc\MSRPV\MSRPV_0173_3_C14.mht) | -0.348 |
| 9 | Pyruvate dehydrogenase E1 component, AceE | **Q59097** | [121](file:///G:\paper-Bcc\PSR\PSR_0173_3_E14.mht) | [3](file:///G:\paper-Bcc\PSR\PSR_0173_3_E14.mht) | [5](file:///G:\paper-Bcc\MSRPV\MSRPV_0173_3_E14.mht) | -0.364 |
| 10 | Aconitate hydratase, AcnA | **Q0BAG3** | [196](file:///G:\paper-Bcc\PSR\PSR_0173_3_G14.mht) | [7](file:///G:\paper-Bcc\PSR\PSR_0173_3_G14.mht) | [7](file:///G:\paper-Bcc\MSRPV\MSRPV_0173_3_G14.mht) | -0.206 |
| 13 | Transketolase 1, TktA | **A2W536** | [193](file:///G:\paper-Bcc\PSR\PSR_0173_3_M14.mht) | [12](file:///G:\paper-Bcc\PSR\PSR_0173_3_M14.mht) | [21](file:///G:\paper-Bcc\MSRPV\MSRPV_0173_3_M14.mht) | -0.117 |
| 15 | Putative biotin carboxylase | **A2W339** | [143](file:///G:\paper-Bcc\PSR\PSR_0173_1_D14.mht) | [5](file:///G:\paper-Bcc\PSR\PSR_0173_1_D14.mht) | [10](file:///G:\paper-Bcc\MSRPV\MSRPV_0173_1_D14.mht) | +0.011 |
| 16 | Oligopeptidase A | **Q1BHV4** | [106](file:///G:\paper-Bcc\PSR\PSR_0173_1_F14.mht) | [7](file:///G:\paper-Bcc\PSR\PSR_0173_1_F14.mht) | [12](file:///G:\paper-Bcc\MSRPV\MSRPV_0173_1_F14.mht) | -0.326 |
| 17 | 30S ribosomal protein S1, RpsA | **B2JF06** | [170](file:///G:\paper-Bcc\PSR\PSR_0173_1_H14.mht) | [8](file:///G:\paper-Bcc\PSR\PSR_0173_1_H14.mht) | [13](file:///G:\paper-Bcc\MSRPV\MSRPV_0173_1_H14.mht) | -0.294 |
| 18 | 30S ribosomal protein S1, RpsA | **B2JF06** | [147](file:///G:\paper-Bcc\PSR\PSR_0173_1_J14.mht) | [12](file:///G:\paper-Bcc\PSR\PSR_0173_1_J14.mht) | [20](file:///G:\paper-Bcc\MSRPV\MSRPV_0173_1_J14.mht) | -0.294 |
| 20 | S-adenosylmethionine synthetase, MetK | **A4JIQ4** | [473](file:///G:\paper-Bcc\PSR\PSR_0173_1_N14.mht) | [14](file:///G:\paper-Bcc\PSR\PSR_0173_1_N14.mht) | [47](file:///G:\paper-Bcc\MSRPV\MSRPV_0173_1_N14.mht) | -0.166 |
| 22 | Elongation factor Tu, Tuf | **Q123F6** | [210](file:///G:\paper-Bcc\PSR\PSR_0173_3_B14.mht) | [5](file:///G:\paper-Bcc\PSR\PSR_0173_3_B14.mht) | [16](file:///G:\paper-Bcc\MSRPV\MSRPV_0173_3_B14.mht) | -0.144 |
| 25 | AhpC/TSA family protein | **Q63T73** | [285](file:///G:\paper-Bcc\PSR\PSR_0173_3_H14.mht) | [6](file:///G:\paper-Bcc\PSR\PSR_0173_3_H14.mht) | [45](file:///G:\paper-Bcc\MSRPV\MSRPV_0173_3_H14.mht) | -0.325 |
| 29 | ATP-dependent Clp protease proteolytic subunit, ClpP | **A9AJR0** | [130](file:///G:\paper-Bcc\PSR\PSR_0173_3_P14.mht) | [7](file:///G:\paper-Bcc\PSR\PSR_0173_3_P14.mht) | [27](file:///G:\paper-Bcc\MSRPV\MSRPV_0173_3_P14.mht) | -0.072 |
| 33 | Phosphoribosylaminoimidazole (AIR) synthetase, PurM | **A2VSP0** | [194](file:///G:\paper-Bcc\PSR\PSR_0173_0_I15.mht) | [10](file:///G:\paper-Bcc\PSR\PSR_0173_0_I15.mht) | [35](file:///G:\paper-Bcc\MSRPV\MSRPV_0173_0_I15.mht) | +0.138 |
| 34 | Probable acetoacetate decarboxylase, Adc | **A2W219** | [129](file:///G:\paper-Bcc\PSR\PSR_0173_0_K15.mht) | [7](file:///G:\paper-Bcc\PSR\PSR_0173_0_K15.mht) | [26](file:///G:\paper-Bcc\MSRPV\MSRPV_0173_0_K15.mht) | -0.136 |
| 35 | D-alanine-D-alanine ligase, Ddl | **A2VT63** | [429](file:///G:\paper-Bcc\PSR\PSR_0173_0_M15.mht) | [9](file:///G:\paper-Bcc\PSR\PSR_0173_0_M15.mht) | [42](file:///G:\paper-Bcc\MSRPV\MSRPV_0173_0_M15.mht) | +0.006 |
| 36 | Acetylglutamate kinase , ArgB | **Q39BX5** | [151](file:///G:\paper-Bcc\PSR\PSR_0173_0_O15.mht) | [3](file:///G:\paper-Bcc\PSR\PSR_0173_0_O15.mht) | [12](file:///G:\paper-Bcc\MSRPV\MSRPV_0173_0_O15.mht) | +0.094 |
| 37 | Acetylglutamate kinase, ArgB | **Q39BX5** | [153](file:///G:\paper-Bcc\PSR\PSR_0173_2_A15.mht) | [2](file:///G:\paper-Bcc\PSR\PSR_0173_2_A15.mht) | [8](file:///G:\paper-Bcc\MSRPV\MSRPV_0173_2_A15.mht) | +0.094 |
| 38 | Electron transfer flavoprotein, alpha subunit, EtfA | **Q1BY10** | [369](file:///G:\paper-Bcc\PSR\PSR_0173_2_C15.mht) | [6](file:///G:\paper-Bcc\PSR\PSR_0173_2_C15.mht) | [38](file:///G:\paper-Bcc\MSRPV\MSRPV_0173_2_C15.mht) | +0.281 |
| 44 | Putative phenylacetic acid degradation enoyl-CoA hydratase, PaaF | **A2VT78** | [207](file:///G:\paper-Bcc\PSR\PSR_0173_0_H15.mht) | [8](file:///G:\paper-Bcc\PSR\PSR_0173_0_H15.mht) | [27](file:///G:\paper-Bcc\MSRPV\MSRPV_0173_0_H15.mht) | +0.074 |
| 47 | KHG/KDPG aldolase, Eda | **A2VSX6** | [168](file:///G:\paper-Bcc\PSR\PSR_0173_2_B15.mht) | [4](file:///G:\paper-Bcc\PSR\PSR_0173_2_B15.mht) | [25](file:///G:\paper-Bcc\MSRPV\MSRPV_0173_2_B15.mht) | +0.310 |
| 52 | ATP phosphoribosyltransferase regulatory subunit, HisZ | **A2VXX6** | [122](file:///G:\paper-Bcc\PSR\PSR_0173_2_N15.mht) | [3](file:///G:\paper-Bcc\PSR\PSR_0173_2_N15.mht) | [24](file:///G:\paper-Bcc\MSRPV\MSRPV_0173_2_N15.mht) | -0.075 |
| 64 | Putative D-beta-hydroxybutyrate dehydrogenase | **A4JKF8** | [171](file:///G:\paper-Bcc\PSR\PSR_0173_3_G15.mht) | [4](file:///G:\paper-Bcc\PSR\PSR_0173_3_G15.mht) | [17](file:///G:\paper-Bcc\MSRPV\MSRPV_0173_3_G15.mht) | +0.149 |
| 68 | Putative siderophore-interacting protein | **B1YXQ3** | [153](file:///G:\paper-Bcc\PSR\PSR_0173_3_O15.mht) | [4](file:///G:\paper-Bcc\PSR\PSR_0173_3_O15.mht) | [13](file:///G:\paper-Bcc\MSRPV\MSRPV_0173_3_O15.mht) | -0.267 |
| 69 | Bifunctional protein FolD | **A0K8R4** | [278](file:///G:\paper-Bcc\PSR\PSR_0173_1_B15.mht) | [8](file:///G:\paper-Bcc\PSR\PSR_0173_1_B15.mht) | [28](file:///G:\paper-Bcc\MSRPV\MSRPV_0173_1_B15.mht) | +0.188 |
| 75 | S-formylglutathione hydrolase | **A2VVV1** | [201](file:///G:\paper-Bcc\PSR\PSR_0173_1_N15.mht) | [6](file:///G:\paper-Bcc\PSR\PSR_0173_1_N15.mht) | [21](file:///G:\paper-Bcc\MSRPV\MSRPV_0173_1_N15.mht) | -0.076 |
| 81 | Putative aldo/keto reductase | **Q0B5L9** | [275](file:///G:\paper-Bcc\PSR\PSR_0173_3_J15.mht) | [4](file:///G:\paper-Bcc\PSR\PSR_0173_3_J15.mht) | [13](file:///G:\paper-Bcc\MSRPV\MSRPV_0173_3_J15.mht) | -0.074 |
| 87 | Glyceraldehyde-3-phosphate dehydrogenase, GapA | **A2VSZ8** | [385](file:///G:\paper-Bcc\PSR\PSR_0174_0_O16.mht) | [8](file:///G:\paper-Bcc\PSR\PSR_0174_0_O16.mht) | [34](file:///G:\paper-Bcc\MSRPV\MSRPV_0174_0_O16.mht) | -0.143 |
| 90 | Putative saccharopine dehydrogenase | **Q1BGN7** | [577](file:///G:\paper-Bcc\PSR\PSR_0174_2_E16.mht) | [9](file:///G:\paper-Bcc\PSR\PSR_0174_2_E16.mht) | [26](file:///G:\paper-Bcc\MSRPV\MSRPV_0174_2_E16.mht) | -0.041 |
| 92 | Fructose-bisphosphate aldolase, CbbA | **A2VVT0** | [745](file:///G:\paper-Bcc\PSR\PSR_0174_2_I16.mht) | [15](file:///G:\paper-Bcc\PSR\PSR_0174_2_I16.mht) | [40](file:///G:\paper-Bcc\MSRPV\MSRPV_0174_2_I16.mht) | -0.197 |
| 96 | Putative saccharopine dehydrogenase | **Q1BGN7** | [329](file:///G:\paper-Bcc\PSR\PSR_0174_0_B16.mht) | [8](file:///G:\paper-Bcc\PSR\PSR_0174_0_B16.mht) | [21](file:///G:\paper-Bcc\MSRPV\MSRPV_0174_0_B16.mht) | -0.041 |
| 105 | Adenylosuccinate synthetase, PurA | **Q39FS0** | [153](file:///G:\paper-Bcc\PSR\PSR_0174_2_H16.mht) | [9](file:///G:\paper-Bcc\PSR\PSR_0174_2_H16.mht) | [22](file:///G:\paper-Bcc\MSRPV\MSRPV_0174_2_H16.mht) | -0.116 |
| 108 | Citrate synthase, GltA | **A2WDT7** | [118](file:///G:\paper-Bcc\PSR\PSR_0174_2_N16.mht) | [7](file:///G:\paper-Bcc\PSR\PSR_0174_2_N16.mht) | [20](file:///G:\paper-Bcc\MSRPV\MSRPV_0174_2_N16.mht) | -0.186 |
| 109 | Dihydrolipoyl dehydrogenase, OdhL | **B1YP46** | [152](file:///G:\paper-Bcc\PSR\PSR_0174_2_P16.mht) | [10](file:///G:\paper-Bcc\PSR\PSR_0174_2_P16.mht) | [29](file:///G:\paper-Bcc\MSRPV\MSRPV_0174_2_P16.mht) | +0.017 |
| 112 | Putative aldehyde dehydrogenase family protein | **A2W309** | [322](file:///G:\paper-Bcc\PSR\PSR_0174_1_E16.mht) | [8](file:///G:\paper-Bcc\PSR\PSR_0174_1_E16.mht) | [23](file:///G:\paper-Bcc\MSRPV\MSRPV_0174_1_E16.mht) | +0.030 |
| 113 | Bifunctional purine biosynthesis protein, PurH | **Q0BI80** | [241](file:///G:\paper-Bcc\PSR\PSR_0174_1_G16.mht) | [10](file:///G:\paper-Bcc\PSR\PSR_0174_1_G16.mht) | [23](file:///G:\paper-Bcc\MSRPV\MSRPV_0174_1_G16.mht) | +0.002 |
| 115 | GMP synthase [glutamine-hydrolyzing], GuaA | **Q1BHF2** | [172](file:///G:\paper-Bcc\PSR\PSR_0174_1_K16.mht) | [11](file:///G:\paper-Bcc\PSR\PSR_0174_1_K16.mht) | [24](file:///G:\paper-Bcc\MSRPV\MSRPV_0174_1_K16.mht) | -0.117 |
| 121 | Inosine-5'-monophosphate dehydrogenase, GuaB | **A4JF47** | [274](file:///G:\paper-Bcc\PSR\PSR_0174_3_I16.mht) | [12](file:///G:\paper-Bcc\PSR\PSR_0174_3_I16.mht) | [33](file:///G:\paper-Bcc\MSRPV\MSRPV_0174_3_I16.mht) | -0.036 |
| 128 | Nematocidal protein AidA | **Q1BJ23** | 241 | 6 | 46 | -0.266 |
| 130 | 60 kDa chaperonin 1, GroEL | Q9ZFE0 | 105 | 7 | 19 | -0.047 |
| 131 | Hypothetical protein | **B1KBA2** | 100 | 6 | 19 | -0.081 |
| 132 | NADH-quinone oxidoreductase | **A2VWQ9** | 86 | 6 | 16 | +0.008 |
| 143 | Putative aminotransferase | **A0AXU0** | [180](file:///G:\paper-Bcc\PSR\PSR_0191_2_L15.mht) | [4](file:///G:\paper-Bcc\PSR\PSR_0191_2_L15.mht) | [13](file:///G:\paper-Bcc\MSRPV\MSRPV_0191_2_L15.mht) | +0.020 |
| 147 | Enolase, Eno | **A4JFY5** | [351](file:///G:\paper-Bcc\PSR\PSR_0191_1_C15.mht) | [9](file:///G:\paper-Bcc\PSR\PSR_0191_1_C15.mht) | [22](file:///G:\paper-Bcc\MSRPV\MSRPV_0191_1_C15.mht) | -0.098 |
| 149 | Putative cyclopropane-fatty-acyl-phospholipid synthase | **A2VVY4** | [221](file:///G:\paper-Bcc\PSR\PSR_0191_1_K15.mht) | [14](file:///G:\paper-Bcc\PSR\PSR_0191_1_K15.mht) | [36](file:///G:\paper-Bcc\MSRPV\MSRPV_0191_1_K15.mht) | -0.306 |
| 153 | Histidinol dehydrogenase, HisD | **A2VTH5** | [269](file:///G:\paper-Bcc\PSR\PSR_0191_3_C15.mht) | [8](file:///G:\paper-Bcc\PSR\PSR_0191_3_C15.mht) | [16](file:///G:\paper-Bcc\MSRPV\MSRPV_0191_3_C15.mht) | +0.012 |
| 154 | Chaperone protein DnaK | **O68191** | [105](file:///G:\paper-Bcc\PSR\PSR_0191_3_E15.mht) | [7](file:///G:\paper-Bcc\PSR\PSR_0191_3_E15.mht) | [12](file:///G:\paper-Bcc\MSRPV\MSRPV_0191_3_E15.mht) | -0.325 |
| 155 | Pyruvate dehydrogenase E1 component, AceE | **Q59097** | [147](file:///G:\paper-Bcc\PSR\PSR_0191_3_G15.mht) | [7](file:///G:\paper-Bcc\PSR\PSR_0191_3_G15.mht) | [9](file:///G:\paper-Bcc\MSRPV\MSRPV_0191_3_G15.mht) | -0.364 |
| 156 | Acetyl-CoA synthetase 1, AcoE | **A2VWW0** | [161](file:///G:\paper-Bcc\PSR\PSR_0191_3_K15.mht) | [7](file:///G:\paper-Bcc\PSR\PSR_0191_3_K15.mht) | [18](file:///G:\paper-Bcc\MSRPV\MSRPV_0191_3_K15.mht) | -0.193 |
| 159 | ClpB heat-shock protein, ClpB | **Q0BET2** | [194](file:///G:\paper-Bcc\PSR\PSR_0191_1_D15.mht) | [14](file:///G:\paper-Bcc\PSR\PSR_0191_1_D15.mht) | [23](file:///G:\paper-Bcc\MSRPV\MSRPV_0191_1_D15.mht) | -0.348 |
| 160 | Pyruvate dehydrogenase E1 component, AceE | **Q59097** | [185](file:///G:\paper-Bcc\PSR\PSR_0191_1_H15.mht) | [7](file:///G:\paper-Bcc\PSR\PSR_0191_1_H15.mht) | [8](file:///G:\paper-Bcc\MSRPV\MSRPV_0191_1_H15.mht) | -0.364 |
| 167 | Acetyl-CoA carboxylase biotin carboxyl carrier protein subunit, AccB | **A2VT29** | [107](file:///G:\paper-Bcc\PSR\PSR_0191_3_J15.mht) | [2](file:///G:\paper-Bcc\PSR\PSR_0191_3_J15.mht) | [26](file:///G:\paper-Bcc\MSRPV\MSRPV_0191_3_J15.mht) | +0.090 |
| 168 | Malate synthase G, GlcB | **Q1BJZ1** | [229](file:///G:\paper-Bcc\PSR\PSR_0191_3_P15.mht) | [9](file:///G:\paper-Bcc\PSR\PSR_0191_3_P15.mht) | [14](file:///G:\paper-Bcc\MSRPV\MSRPV_0191_3_P15.mht) | -0.166 |
| 170 | Phosphoenolpyruvate carboxykinase, PckG | **Q1BNJ4** | 174 | 12 | 20 | -0.268 |
| 171 | Phosphoenolpyruvate carboxykinase, PckG | **Q1BNJ4** | [361](file:///G:\paper-Bcc\PSR\PSR_0192_2_I16.mht) | [13](file:///G:\paper-Bcc\PSR\PSR_0192_2_I16.mht) | [21](file:///G:\paper-Bcc\MSRPV\MSRPV_0192_2_I16.mht) | -0.268 |
| 176 | Trigger factor, Tig | **Q0BEF5** | [149](file:///G:\paper-Bcc\PSR\PSR_0192_0_J16.mht) | [10](file:///G:\paper-Bcc\PSR\PSR_0192_0_J16.mht) | [22](file:///G:\paper-Bcc\MSRPV\MSRPV_0192_0_J16.mht) | -0.466 |
| 177 | 30S ribosomal protein S1, RpsA | B2JF06 | [122](file:///G:\paper-Bcc\PSR\PSR_0192_0_L16.mht) | [7](file:///G:\paper-Bcc\PSR\PSR_0192_0_L16.mht) | [11](file:///G:\paper-Bcc\MSRPV\MSRPV_0192_0_L16.mht) | -0.294 |
| 178 | Alanine-tRNA ligase, AlaS | **Q1BX19** | [216](file:///G:\paper-Bcc\PSR\PSR_0192_man_0_N16.mht) | [15](file:///G:\paper-Bcc\PSR\PSR_0192_man_0_N16.mht) | [20](file:///G:\paper-Bcc\MSRPV\MSRPV_0192_man_0_N16.mht) | -0.286 |
| 181 | Arginine-tRNA ligase, ArgS | **A2VV06** | [493](file:///G:\paper-Bcc\PSR\PSR_0192_2_D16.mht) | [13](file:///G:\paper-Bcc\PSR\PSR_0192_2_D16.mht) | [31](file:///G:\paper-Bcc\MSRPV\MSRPV_0192_2_D16.mht) | -0.172 |
| 183 | Glutamyl-tRNA(Gln) amidotransferase subunit A, GatA | **Q1BSL2** | [217](file:///G:\paper-Bcc\PSR\PSR_0192_2_H16.mht) | [9](file:///G:\paper-Bcc\PSR\PSR_0192_2_H16.mht) | [24](file:///G:\paper-Bcc\MSRPV\MSRPV_0192_2_H16.mht) | -0.087 |
| 186 | Elongation factor G1, FusA | **Q1BRU5** | [142](file:///G:\paper-Bcc\PSR\PSR_0192_2_P16.mht) | [9](file:///G:\paper-Bcc\PSR\PSR_0192_2_P16.mht) | [16](file:///G:\paper-Bcc\MSRPV\MSRPV_0192_2_P16.mht) | -0.328 |
| 187 | Isoleucine-tRNA ligase, IleS | **A4JH32** | [195](file:///G:\paper-Bcc\PSR\PSR_0192_1_A16.mht) | [11](file:///G:\paper-Bcc\PSR\PSR_0192_1_A16.mht) | [11](file:///G:\paper-Bcc\MSRPV\MSRPV_0192_1_A16.mht) | -0.349 |
| 189 | Dihydrolipoyl dehydrogenase, OdhL | **B1YP46** | [324](file:///G:\paper-Bcc\PSR\PSR_0192_1_E16.mht) | [7](file:///G:\paper-Bcc\PSR\PSR_0192_1_E16.mht) | [11](file:///G:\paper-Bcc\MSRPV\MSRPV_0192_1_E16.mht) | +0.017 |
| 190 | Elongation factor Ts, Tsf | **Q0BE16** | [683](file:///G:\paper-Bcc\PSR\PSR_0192_1_G16.mht) | [13](file:///G:\paper-Bcc\PSR\PSR_0192_1_G16.mht) | [39](file:///G:\paper-Bcc\MSRPV\MSRPV_0192_1_G16.mht) | +0.048 |
| 198 | (3R)-hydroxymyristoyl-(acyl carrier protein) dehydratase, FabZ | **Q13XC7** | [115](file:///G:\paper-Bcc\PSR\PSR_0192_3_I16.mht) | [2](file:///G:\paper-Bcc\PSR\PSR_0192_3_I16.mht) | [10](file:///G:\paper-Bcc\MSRPV\MSRPV_0192_3_I16.mht) | +0.036 |
| 201 | Outer membrane protein assembly factor YaeT | **A2VXD7** | [572](file:///G:\paper-Bcc\PSR\PSR_0204_0_O15.mht) | [26](file:///G:\paper-Bcc\PSR\PSR_0204_0_O15.mht) | [45](file:///G:\paper-Bcc\MSRPV\MSRPV_0204_0_O15.mht) | -0.308 |
| 202 | Putative pyochelin receptor protein FptA | **A2W3M3** | [739](file:///G:\paper-Bcc\PSR\PSR_0204_2_A15.mht) | [22](file:///G:\paper-Bcc\PSR\PSR_0204_2_A15.mht) | [31](file:///G:\paper-Bcc\MSRPV\MSRPV_0204_2_A15.mht) | -0.292 |
| 203 | TonB-dependent receptor | **Q1BM21** | [423](file:///G:\paper-Bcc\PSR\PSR_0204_2_C15.mht) | [20](file:///G:\paper-Bcc\PSR\PSR_0204_2_C15.mht) | [37](file:///G:\paper-Bcc\MSRPV\MSRPV_0204_2_C15.mht) | -0.330 |
| 204 | Succinate dehydrogenase flavoprotein subunit, SdhA | **A2VZR7** | [329](file:///G:\paper-Bcc\PSR\PSR_0204_2_E15.mht) | [17](file:///G:\paper-Bcc\PSR\PSR_0204_2_E15.mht) | [35](file:///G:\paper-Bcc\MSRPV\MSRPV_0204_2_E15.mht) | -0.306 |
| 205 | Succinate dehydrogenase flavoprotein subunit, SdhA | **A2VZR7** | [612](file:///G:\paper-Bcc\PSR\PSR_0204_2_G15.mht) | [18](file:///G:\paper-Bcc\PSR\PSR_0204_2_G15.mht) | [44](file:///G:\paper-Bcc\MSRPV\MSRPV_0204_2_G15.mht) | -0.306 |
| 207 | Hypothetical protein | **B1KBA2** | [303](file:///G:\paper-Bcc\PSR\PSR_0204_2_K15.mht) | [7](file:///G:\paper-Bcc\PSR\PSR_0204_2_K15.mht) | [45](file:///G:\paper-Bcc\MSRPV\MSRPV_0204_2_K15.mht) | -0.081 |
| 212 | Putative outer membrane protein | **A2VQ60** | [504](file:///G:\paper-Bcc\PSR\PSR_0204_0_F15.mht) | [10](file:///G:\paper-Bcc\PSR\PSR_0204_0_F15.mht) | [55](file:///G:\paper-Bcc\MSRPV\MSRPV_0204_0_F15.mht) | +0.119 |
| 213 | Putrescine ABC transporter ATP-binding protein, PotG | **Q1BGR3** | [139](file:///G:\paper-Bcc\PSR\PSR_0204_0_H15.mht) | [8](file:///G:\paper-Bcc\PSR\PSR_0204_0_H15.mht) | [26](file:///G:\paper-Bcc\MSRPV\MSRPV_0204_0_H15.mht) | -0.087 |
| 215 | Putative acetyl-CoA synthetase | **A2W2F4** | [273](file:///G:\paper-Bcc\PSR\PSR_0204_0_L15.mht) | [13](file:///G:\paper-Bcc\PSR\PSR_0204_0_L15.mht) | [18](file:///G:\paper-Bcc\MSRPV\MSRPV_0204_0_L15.mht) | +0.033 |
| 216 | Putative dehydrogenase, zinc-binding subunit | **B1K5B2** | [152](file:///G:\paper-Bcc\PSR\PSR_0204_0_N15.mht) | [4](file:///G:\paper-Bcc\PSR\PSR_0204_0_N15.mht) | [19](file:///G:\paper-Bcc\MSRPV\MSRPV_0204_0_N15.mht) | +0.097 |
| 220 | DNA polymerase I, PolA | **Q1BLY0** | [469](file:///G:\paper-Bcc\PSR\PSR_0204_2_J15.mht) | [19](file:///G:\paper-Bcc\PSR\PSR_0204_2_J15.mht) | [22](file:///G:\paper-Bcc\MSRPV\MSRPV_0204_2_J15.mht) | -0.224 |
| 227 | ATP synthase subunit alpha, AtpA | **A2VTZ2** | [546](file:///G:\paper-Bcc\PSR\PSR_0204_1_O15.mht) | [20](file:///G:\paper-Bcc\PSR\PSR_0204_1_O15.mht) | [44](file:///G:\paper-Bcc\MSRPV\MSRPV_0204_1_O15.mht) | -0.142 |
| 229 | Isocitrate lyase | **Q1BNJ5** | [175](file:///G:\paper-Bcc\PSR\PSR_0204_3_C15.mht) | [11](file:///G:\paper-Bcc\PSR\PSR_0204_3_C15.mht) | [21](file:///G:\paper-Bcc\MSRPV\MSRPV_0204_3_C15.mht) | -0.359 |
| 231 | Putative amino acid ABC transporter ATP-binding protein, LivF | **A2W3K2** | [217](file:///G:\paper-Bcc\PSR\PSR_0204_3_G15.mht) | [11](file:///G:\paper-Bcc\PSR\PSR_0204_3_G15.mht) | [48](file:///G:\paper-Bcc\MSRPV\MSRPV_0204_3_G15.mht) | +0.080 |
| 232 | Hypothetical protein | **A2VW75** | [198](file:///G:\paper-Bcc\PSR\PSR_0204_3_I15.mht) | [5](file:///G:\paper-Bcc\PSR\PSR_0204_3_I15.mht) | [21](file:///G:\paper-Bcc\MSRPV\MSRPV_0204_3_I15.mht) | -0.086 |
| 233 | Putative exported isomerase | **Q1BH74** | [246](file:///G:\paper-Bcc\PSR\PSR_0204_3_K15.mht) | [13](file:///G:\paper-Bcc\PSR\PSR_0204_3_K15.mht) | [47](file:///G:\paper-Bcc\MSRPV\MSRPV_0204_3_K15.mht) | -0.437 |
| 235 | ABC transporter related (Polar amino acid transport) | **A9AH88** | [102](file:///G:\paper-Bcc\PSR\PSR_0204_3_O15.mht) | [6](file:///G:\paper-Bcc\PSR\PSR_0204_3_O15.mht) | [30](file:///G:\paper-Bcc\MSRPV\MSRPV_0204_3_O15.mht) | -0.173 |
| 236 | Putative N-methylproline demethylase | **A2WG89** | [160](file:///G:\paper-Bcc\PSR\PSR_0204_1_D15.mht) | [10](file:///G:\paper-Bcc\PSR\PSR_0204_1_D15.mht) | [17](file:///G:\paper-Bcc\MSRPV\MSRPV_0204_1_D15.mht) | -0.216 |
| 238 | Succinate dehydrogenase flavoprotein subunit, SdhA | **A2VZR7** | [259](file:///G:\paper-Bcc\PSR\PSR_0204_1_H15.mht) | [19](file:///G:\paper-Bcc\PSR\PSR_0204_1_H15.mht) | [40](file:///G:\paper-Bcc\MSRPV\MSRPV_0204_1_H15.mht) | -0.306 |
| 240 | Putative iron transport-related ATP-binding protein, OrbC | **Q1BWE3** | [161](file:///G:\paper-Bcc\PSR\PSR_0204_1_L15.mht) | [4](file:///G:\paper-Bcc\PSR\PSR_0204_1_L15.mht) | [17](file:///G:\paper-Bcc\MSRPV\MSRPV_0204_1_L15.mht) | +0.042 |
| 242 | Outer membrane protein assembly factor YaeT | **A2W9B0** | [314](file:///G:\paper-Bcc\PSR\PSR_0204_1_P15.mht) | [14](file:///G:\paper-Bcc\PSR\PSR_0204_1_P15.mht) | [19](file:///G:\paper-Bcc\MSRPV\MSRPV_0204_1_P15.mht) | -0.308 |
| 243 | Putative prolyl oligopeptidase | **Q1BHV9** | [170](file:///G:\paper-Bcc\PSR\PSR_0204_3_D15.mht) | [12](file:///G:\paper-Bcc\PSR\PSR_0204_3_D15.mht) | [14](file:///G:\paper-Bcc\MSRPV\MSRPV_0204_3_D15.mht) | -0.431 |
| 248 | Two-component regulatory system, response regulator protein | **Q0BAX7** | [190](file:///G:\paper-Bcc\PSR\PSR_0204_3_P15.mht) | [5](file:///G:\paper-Bcc\PSR\PSR_0204_3_P15.mht) | [32](file:///G:\paper-Bcc\MSRPV\MSRPV_0204_3_P15.mht) | -0.172 |
| 250 | Hypothetical protein | **A2VS76** | [162](file:///G:\paper-Bcc\PSR\PSR_0205_0_I19.mht) | [3](file:///G:\paper-Bcc\PSR\PSR_0205_0_I19.mht) | [20](file:///G:\paper-Bcc\MSRPV\MSRPV_0205_0_I19.mht) | -0.030 |
| 252 | Putative phosphorous metabolism-related protein | **A2VXR9** | [147](file:///G:\paper-Bcc\PSR\PSR_0205_2_C19.mht) | [7](file:///G:\paper-Bcc\PSR\PSR_0205_2_C19.mht) | [14](file:///G:\paper-Bcc\MSRPV\MSRPV_0205_2_C19.mht) | -0.347 |
| 253 | NADP-dependent malic enzyme | **Q1BRR9** | [225](file:///G:\paper-Bcc\PSR\PSR_0205_2_E19.mht) | [10](file:///G:\paper-Bcc\PSR\PSR_0205_2_E19.mht) | [15](file:///G:\paper-Bcc\MSRPV\MSRPV_0205_2_E19.mht) | +0.052 |
| 254 | Transketolase 1, TktA | **A2W536** | [256](file:///G:\paper-Bcc\PSR\PSR_0205_2_G19.mht) | [9](file:///G:\paper-Bcc\PSR\PSR_0205_2_G19.mht) | [15](file:///G:\paper-Bcc\MSRPV\MSRPV_0205_2_G19.mht) | -0.117 |
| 255 | 5,10-methylenetetrahydrofolate reductase, MetF | **A2VU73** | [215](file:///G:\paper-Bcc\PSR\PSR_0205_2_I19.mht) | [7](file:///G:\paper-Bcc\PSR\PSR_0205_2_I19.mht) | [28](file:///G:\paper-Bcc\MSRPV\MSRPV_0205_2_I19.mht) | -0.143 |
| 256 | Putative uncharacterized protein | **Q1BT08** | [157](file:///G:\paper-Bcc\PSR\PSR_0205_2_K19.mht) | [4](file:///G:\paper-Bcc\PSR\PSR_0205_2_K19.mht) | [18](file:///G:\paper-Bcc\MSRPV\MSRPV_0205_2_K19.mht) | +0.242 |
| 257 | Electron transfer flavoprotein, alpha subunit, EtfA | **Q1BY10** | [611](file:///G:\paper-Bcc\PSR\PSR_0205_2_M19.mht) | [7](file:///G:\paper-Bcc\PSR\PSR_0205_2_M19.mht) | [34](file:///G:\paper-Bcc\MSRPV\MSRPV_0205_2_M19.mht) | +0.281 |
| 258 | Transcription antitermination protein NusG | **Q39KI0** | [170](file:///G:\paper-Bcc\PSR\PSR_0205_2_O19.mht) | [5](file:///G:\paper-Bcc\PSR\PSR_0205_2_O19.mht) | [35](file:///G:\paper-Bcc\MSRPV\MSRPV_0205_2_O19.mht) | -0.521 |
| 261 | Fumarate hydratase class II, FumC | **A2WBM9** | [287](file:///G:\paper-Bcc\PSR\PSR_0205_0_J19.mht) | [11](file:///G:\paper-Bcc\PSR\PSR_0205_0_J19.mht) | [27](file:///G:\paper-Bcc\MSRPV\MSRPV_0205_0_J19.mht) | -0.048 |
| 262 | Putative aldehyde dehydrogenase | **Q1BNF8** | [148](file:///G:\paper-Bcc\PSR\PSR_0205_0_L19.mht) | [8](file:///G:\paper-Bcc\PSR\PSR_0205_0_L19.mht) | [25](file:///G:\paper-Bcc\MSRPV\MSRPV_0205_0_L19.mht) | +0.039 |
| 263 | Hypothetical protein | **Q1BIT4** | [167](file:///G:\paper-Bcc\PSR\PSR_0205_0_N19.mht) | [6](file:///G:\paper-Bcc\PSR\PSR_0205_0_N19.mht) | [27](file:///G:\paper-Bcc\MSRPV\MSRPV_0205_0_N19.mht) | +0.118 |
| 266 | Lectin (Fucose-binding lectin II), BclB | **Q1BKJ6** | 142 | 2 | 12 | -0.064 |
| 267 | Phosphomannomutase, ManB | **B1JX22** | [468](file:///G:\paper-Bcc\PSR\PSR_0205_1_C19.mht) | [14](file:///G:\paper-Bcc\PSR\PSR_0205_1_C19.mht) | [34](file:///G:\paper-Bcc\MSRPV\MSRPV_0205_1_C19.mht) | -0.169 |
| 269 | NAD-dependent epimerase/dehydratase | **A4JCW7** | [135](file:///G:\paper-Bcc\PSR\PSR_0205_1_G19.mht) | [7](file:///G:\paper-Bcc\PSR\PSR_0205_1_G19.mht) | [23](file:///G:\paper-Bcc\MSRPV\MSRPV_0205_1_G19.mht) | -0.047 |
| 270 | Serine hydroxymethyltransferase, GlyA | **A2VSL1** | [196](file:///G:\paper-Bcc\PSR\PSR_0205_1_I19.mht) | [6](file:///G:\paper-Bcc\PSR\PSR_0205_1_I19.mht) | [18](file:///G:\paper-Bcc\MSRPV\MSRPV_0205_1_I19.mht) | -0.180 |
| 272 | 2-methylcitrate synthase, PrpC | **Q1BJI6** | [291](file:///G:\paper-Bcc\PSR\PSR_0205_1_M19.mht) | [11](file:///G:\paper-Bcc\PSR\PSR_0205_1_M19.mht) | [37](file:///G:\paper-Bcc\MSRPV\MSRPV_0205_1_M19.mht) | -0.217 |
| 276 | Phosphoenolpyruvate synthase, PpsA | **A9AIN1** | 231 | [9](file:///G:\paper-Bcc\PSR\PSR_0173_1_M14.mht) | [14](file:///G:\paper-Bcc\MSRPV\MSRPV_0173_1_M14.mht) | -0.218 |
| 277 | Putative ornithine decarboxylase | **A4JGW1** | 502 | [16](file:///G:\paper-Bcc\PSR\PSR_0173_1_O14.mht) | [25](file:///G:\paper-Bcc\MSRPV\MSRPV_0173_1_O14.mht) | -0.206 |

*^a^* The GRAVY index was calculated using the online tool ProtParam (http://web.expasy.org/protparam/). Proteins where the GRAVY index is underlined represent cases where, although the calculated GRAVY index is negative (i.e. hydrophilic), the protein also possesses a highly hydrophobic domain (typically indicative of a membrane-binding domain).
