# Supplementary material for: Proteomic Profiling of Burkholderia cenocepacia Clonal Isolates with Different Virulence Potential Retrieved from a Cystic Fibrosis Patient during Chronic Lung Infection
Source: PLoS One. 2013 Dec 13;8(12):e83065. doi: 10.1371/journal.pone.0083065 (PMC3862766; doi:10.1371/journal.pone.0083065)
Supplement: Table S2 — Proteins whose content was found to be increased or decreased in isolates IST4113 and 4134, compared to IST439, and differently expressed, in the same direction, in cells of B. cenocepacia J2315 in response to oxygen limitation, using the corresponding microarray dataset published in Sass et al. (2013)*. A fold-change cut-off of ±1.5 was used. (DOCX) [file pone.0083065.s002.docx]

**Table S2** – Proteins whose content was found to be increased or decreased in isolates IST4113 and 4134, compared to IST439, and differently expressed, in the same direction, in cells of *B. cenocepacia* J2315 in response to oxygen limitation, using the corresponding microarray dataset published in Sass *et al.* (2013)*. A fold-change cut-off of ±1.5 was used.

|  |  | Fold-Change | |  |
| --- | --- | --- | --- | --- |
| Gene ID | Gene name and  Protein function | 4113  439 | 4134  439 | Microarray  Fold-change*  6% O_2_  21% O_2_ |
| *Protein folding* | | | | |
| *BCAL1919* | *clpB*: ClpB heat-shock protein | 6.0 | 2.7 | 15.5 |
| *BCAL1919* | *clpB*: ClpB heat-shock protein | 5.4 | 2.8 |  |
| *BCAL1996* | *clpP*: ATP-dependent Clp protease proteolytic subunit | 0.7 | 0.9 | 0.6 |
| *BCAL3146* | *groEL*: 60 kDa chaperonin 1 | 1.7 | 1.2 | 2.3 |
| *Transcription* | | | | |
| *BCAL0221* | *nusG*: Transcription antitermination protein NusG | 0.6 | 0.6 | 0.5 |
| *Translation* | | | | |
| *BCAL2213* | Oligopeptidase A | 2.5 | 1.4 | 1.9 |
| *Amino acid metabolism* | | | | |
| *BCAL2221* | Putative prolyl oligopeptidase | 1.2 | 2.7 | 2.0 |
| *BCAL3197* | *glyA1*: Serine hydroxymethyltransferase | 0.7 | 0.9 | 0.4 |
| *Cell envelope biogenesis* | | | | |
| *BCAL0409* | *paaF*: Putative phenylacetic acid degradation enoyl-CoA hydratase PaaF | 0.7 | 0.8 | 0.5 |
| *BCAL2783* | Putative cyclopropane-fatty-acyl-phospholipid synthase | 3.1 | 1.5 | 7.1 |
| *BCAL3420* | *accB*: Acetyl-CoA carboxylase biotin carboxyl carrier protein subunit | 0.6 | 0.8 | 0.3 |
| *BCAL2080* | *fabZ*: (3R)-hydroxymyristoyl-(acyl carrier protein) dehydratase | 0.7 | 0.9 | 0.5 |
| *BCAL1829* | Putative outer membrane protein | 1.6 | 2.0 | 240.5 |
| *Energy metabolism* | | | | |
| *BCAL2209* | *aceE*: Pyruvate dehydrogenase E1 component | 3.5 | 2.0 | 2.1 |
| *BCAL2209* | *aceE*: Pyruvate dehydrogenase E1 component | 3.4 | 1.9 |  |
| *BCAL2209* | *aceE*: Pyruvate dehydrogenase E1 component | 3.4 | 1.8 |  |
| *BCAL3389* | *tktA*: Transketolase 1 | 1.5 | 1.3 | 1.7 |
| *BCAM0042* | Putative aldo/keto reductase | 2.3 | 1.4 | 1.9 |
| *BCAM2468* | Putative aldehyde dehydrogenase family protein | 3.4 | 2.1 | 10.2 |
| *BCAM1581* | *pckG*: Phosphoenolpyruvate carboxykinase | 3.8 | 2.2 | 16.0 |
| *BCAM1581* | *pckG*: Phosphoenolpyruvate carboxykinase | 3.9 | 2.2 |  |
| *BCAM2710* | Putative acetyl-CoA synthetase | 4.7 | 1.5 | 10.4 |
| *BCAL0205* | NADP-dependent malic enzyme | 0.3 | 0.5 | 0.2 |
| *Coenzyme metabolism* | | | | |
| *BCAL2212* | *folD*: Bifunctional protein FolD | 1.6 | 1.4 | 1.6 |
| *Transport of small molecules* | | | | |
| *BCAS0242* | Hypothetical protein | 0.6 | 0.6 | 0.7 |
| *Unknown* | | | | |
| *BCAS0293* | *aidA*: Nematocidal protein AidA | 3.0 | 3.2 | 31.0 |
| *BCAL3052* | Hypothetical protein | 0.8 | 0.7 | 0.5 |

* Sass AM, Schmerk C, Agnoli K, Norville PJ, Eberl L, et al. (2013) The unexpected discovery of a novel low-oxygen-activated locus for the anoxic persistence of *Burkholderia cenocepacia*. ISME J.
